# Frequency-Domain Models for Nonlinear Microwave Devices Based on Large-Signal Measurements

**DOI:** 10.6028/jres.109.029

**Published:** 2004-08-01

**Authors:** Jeffrey A. Jargon, Donald C. DeGroot, K. C. Gupta

**Affiliations:** National Institute of Standards and Technology, 325 Broadway, Boulder, CO 80305; Center for Advanced Manufacturing and Packaging of Microwave, Optical, and Digital Electronics (CAMPmode) University of Colorado at Boulder, Boulder, CO 80309

**Keywords:** frequency-domain, large-signal, measurement, microwave, model, network analyzer, nonlinear, scattering parameter

## Abstract

In this paper, we introduce nonlinear large-signal scattering ( S) parameters, a new type of frequency-domain mapping that relates incident and reflected signals. We present a general form of nonlinear large-signal S-parameters and show that they reduce to classic S-parameters in the absence of nonlinearities. Nonlinear large-signal impedance ( Z) and admittance ( D) parameters are also introduced, and equations relating the different representations are derived. We illustrate how nonlinear large-signal S-parameters can be used as a tool in the design process of a nonlinear circuit, specifically a single-diode 1 GHz frequency-doubler. For the case where a nonlinear model is not readily available, we developed a method of extracting nonlinear large-signal S-parameters obtained with artificial neural network models trained with multiple measurements made by a nonlinear vector network analyzer equipped with two sources. Finally, nonlinear large-signal S-parameters are compared to another form of nonlinear mapping, known as nonlinear scattering functions. The nonlinear large-signal S-parameters are shown to be more general.

## 1. Introduction

Vector network analyzers (VNAs) are one of the most versatile instruments available for RF and microwave measurements. They are used to measure complex scattering parameters (*S*-parameters) of linear devices or circuits. RF engineers use them to verify their designs, confirm proper performance, and diagnose failures. A VNA works by exciting a linear device under test (DUT) with a series of sine wave signals, one frequency at a time, and detecting the response of the DUT at its signal ports. Since the DUT is linear, the input and output signal frequencies are the same as the source; these signals can be described by complex numbers that account for the signals’ amplitudes and phases. The input-output relationships are described by ratios of complex numbers, known as *S*-parameters. For a two-port network, four *S*-parameters completely describe the behavior of a linear DUT when excited by a sine wave at a particular frequency. Although the measurement of *S*-parameters by VNAs is invaluable to the microwave designer for modeling and measuring linear circuits, these measurements are oftentimes inadequate for nonlinear circuits operating at large-signal conditions, since nonlinearities transfer energy from the stimulus frequency to products at new frequencies. Thus, conventional linear network analysis, which relies on the assumption of superposition, must be replaced by a more general type of analysis, which we refer to as nonlinear network analysis.

Nonlinear network analysis involves characterizing a nonlinear device under realistic, large-signal operating conditions. To do this, complex traveling waves (rather than ratios) are measured at the ports of a DUT not only at the stimulus frequency (or frequencies), but also at other frequencies where energy may be created. Assuming the input signals are sine-waves and the DUT exhibits neither sub-harmonic nor chaotic behavior, the input and output signals will be combinations of sine-wave signals, caused by the nonlinearity of the DUT in conjunction with impedance mismatches between the measuring system and the DUT. If a single excitation frequency is present, new frequency components will appear at harmonics of the excitation frequency, and if multiple excitation frequencies are present, new frequency components will appear at the intermodulation products as well as at harmonics of each of the excitation frequencies. In practice, there will be a limited number of significant harmonics and intermodulation products. The set of frequencies at which energy is present and must be measured is known as the frequency grid.

A class of instruments known as nonlinear vector network analyzers (NVNA) are capable of providing accurate waveform vectors by acquiring and correcting the magnitude and phase relationships between the fundamental and harmonic components in the periodic signals [1–5]. An NVNA excites a nonlinear DUT with one or more sine wave signals and detects the response of the DUT at its signal ports. Assuming the DUT does not exhibit any sub-harmonic or chaotic behavior, the input and output signals will be combinations of sine wave signals due to the nonlinearity of the DUT in conjunction with mismatches between the system and the DUT. With these facts in mind, the major difference between a linear VNA and an NVNA is that a VNA measures ratios between input and output waves one frequency at a time while an NVNA measures the actual input and output waves simultaneously over a broad band of frequencies.

Even though *S*-parameters cannot adequately represent nonlinear circuits, some type of parameters relating incident and reflected signals are beneficial so that the designers can “see” application-specific engineering figures of merit that are similar to what they are accustomed to. In first part of this paper, we propose definitions of such ratios that we refer to as nonlinear large-signal scattering ( S) parameters. We also introduce nonlinear large-signal impedance ( Z) and admittance ( D) parameters, and present equations relating the different representations. Next, we make two simplifications when considering the cases of a one-port network with a single-tone excitation and a two-port network with a single-tone excitation.

For existing nonlinear models, we can readily generate nonlinear large-signal S-parameters by performing a harmonic balance simulation. For devices, with no model available, we can extract these parameters from artificial neural network (ANN) models that are trained with multiple frequency-domain measurements made on a nonlinear DUT with an NVNA. To illustrate applications and generation of nonlinear large-signal S-parameters, we present two examples. First, we illustrate how nonlinear large-signal S-parameters can be used as a tool in the process of designing a simple nonlinear circuit, specifically a single-diode 1 GHz frequency-doubler circuit. And secondly, we describe a method for generating nonlinear large-signal S-parameters based upon ANN models trained on frequency-domain data measured using an NVNA. We compare a diode circuit model, generated using this method, to a harmonic balance simulation of a commercial device model.

Finally, we compare our nonlinear large-signal S-parameters to another form of nonlinear mapping, known as nonlinear scattering functions [6–7]. Specifically, we show that the two formulations are not equivalent. Nonlinear large-signal S-parameters are more general than the nonlinear scattering functions, which are useful in approximating a specific class of nonlinearity in a more compact form.

## 2. Nonlinear Large-Signal Scattering Parameters

In this section, we introduce the concept of nonlinear large-signal scattering parameters. Like commonly used linear *S*-parameters, nonlinear large-signal scattering ( S) parameters can also be expressed as ratios of incident and reflected wave variables. However, unlike linear *S*-parameters, nonlinear large-signal S-parameters depend upon the signal magnitude and must account for the harmonic content of the input and output signals since energy can be transferred to other frequencies in a nonlinear device.

After presenting the general form of nonlinear large-signal S-parameters, we also introduce nonlinear large-signal impedance ( Z) and admittance ( D) parameters, and present equations for relating the different representations. Next, we make two simplifications in which we consider the cases of a one-port network with a single-tone excitation and a two-port network with a single-tone excitation.

### 2.1 General Form

Consider an *N*-port network. Normalized wave variables *a_jl_* and *b_jl_* at the *j*th port and *l*th harmonic are proportional to the incoming and outgoing waves, respectively, and may be defined in terms of the voltages associated with these waves as follows:

$$a_{jl}=\frac{V_{jl}^{+}}{\sqrt{Z_{oj}}};\;b_{jl}=\frac{V_{jl}^{-}}{\sqrt{Z_{oj}}},$$ (1)

where $V_{jl}^{+}$ and $V_{jl}^{-}$ represent voltages associated with the incoming and outgoing waves in the transmission lines connected to the *j*th port and containing frequencies of the *l*th harmonic; *Z_oj_* represents the characteristic impedance of the line at the *j*th port.

The nonlinear large-signal scattering matrix S of the network expresses the relationship between *a*’s and *b*’s at various ports and harmonics through the matrix equation

b=Sa, (2)

where ***b*** and ***a*** are (*N×M*)-element column vectors. Here *N* refers to the number of ports and *M* refers to the number of harmonics being considered. Matrix S is an (*N×M*)^2^-element square matrix. We assume all *a*’s and *b*’s are phase referenced to *a*_11_ to enforce time invariance [8].

As an example, consider a two-port network with 3 harmonics; Eq. (2) then becomes

$$\left[\frac{{\bar{b}}_{1}}{{\bar{b}}_{2}}\right]=\left[\begin{array}{cc} \left[S_{11}\right] & \left[S_{12}\right]\\ \left[S_{21}\right] & \left[S_{22}\right] \end{array}\right]\;\left[\begin{array}{c} {\bar{a}}_{1}\\ {\bar{a}}_{2} \end{array}\right],$$ (3)

where

$$\left[S_{ij}\right]=\left[\begin{array}{ccc} S_{ij11} & S_{ij12} & S_{ij13}\\ S_{ij21} & S_{ij22} & S_{ij23}\\ S_{ij31} & S_{ij32} & S_{ij33} \end{array}\right].$$ (4)

For each nonlinear large-signal scattering parameter $S_{ijkl}$ the index *i* refers to the port number of the *b* wave, the index *j* refers to the port number of the *a* wave, *k* is the harmonic index of the *b* wave, and *l* is the harmonic index of the *a* wave. The vectors ${\bar{a}}_{j}$ and ${\bar{b}}_{i}$ are (*M*=3)-element vectors given by

$${\bar{a}}_{j}=\left[\begin{array}{c} a_{j1}\\ a_{j2}\\ a_{j3} \end{array}\right];\;{\bar{b}}_{j}=\left[\begin{array}{c} b_{i1}\\ b_{i2}\\ b_{i3} \end{array}\right].$$ (5)

Equation (3) can be expanded as follows

$$\left[\begin{array}{l} b_{11}\\ b_{12}\\ b_{13}\\ b_{21}\\ b_{22}\\ b_{23} \end{array}\right]=\left[\begin{array}{llllll} S_{1111} & S_{1112} & S_{1113} & S_{1211} & S_{1212} & S_{1213}\\ S_{1121} & S_{1122} & S_{1123} & S_{1221} & S_{1222} & S_{1223}\\ S_{1131} & S_{1132} & S_{1133} & S_{1231} & S_{1232} & S_{1233}\\ S_{2111} & S_{2112} & S_{2113} & S_{2211} & S_{2212} & S_{2213}\\ S_{2121} & S_{2122} & S_{2123} & S_{2221} & S_{2222} & S_{2223}\\ S_{2131} & S_{2132} & S_{2133} & S_{2231} & S_{2232} & S_{2233} \end{array}\right]\;[\begin{array}{l} a_{11}\\ a_{12}\\ a_{13}\\ a_{21}\\ a_{22}\\ a_{23} \end{array}].$$ (6)

Note that in each of the four sub-matrices, the diagonal elements contain the same-frequency scattering parameters, the upper right elements contain the frequency down-conversion scattering parameters, and the lower left elements contain the frequency up-conversion scattering parameters. If the device under consideration contains no nonlinearities (i.e., no power is transferred to other frequencies), then Eq. (6) reduces to

$$\left[\begin{array}{c} b_{11}\\ b_{12}\\ b_{13}\\ b_{21}\\ b_{22}\\ b_{23} \end{array}\right]=\left[\begin{array}{cccccc} S_{1111} & 0 & 0 & S_{1211} & 0 & 0\\ 0 & S_{1122} & 0 & 0 & S_{1222} & 0\\ 0 & 0 & S_{1133} & 0 & 0 & S_{1233}\\ S_{2111} & 0 & 0 & S_{2211} & 0 & 0\\ 0 & S_{2122} & 0 & 0 & S_{2222} & 0\\ 0 & 0 & S_{2133} & 0 & 0 & S_{2233} \end{array}\right]\;\left[\begin{array}{c} a_{11}\\ a_{12}\\ a_{13}\\ a_{21}\\ a_{22}\\ a_{23} \end{array}\right]$$ (7)

which is the matrix representation for the well-known linear *S*-parameters involving three excitation frequencies.

### 2.2 Nonlinear Large-Signal Impedance Parameters

Rather than expressing the relationship between *a*’s and *b*’s in terms of a nonlinear large-signal scattering matrix S, we can alternatively express the relationship between voltages (*V*’s) and currents (*I*’s) in terms of a nonlinear large-signal impedance matrix Z, as follows

V=ZI, (8)

where ***V*** and ***I*** are (*N×M*)-element column vectors. Once again *N* refers to the number of ports and *M* refers to the number of harmonics being considered. Z is an (*N×M*)^2^-element square matrix.

For a two-port network with 3 harmonics, Eq. (8) becomes

$$\left[\begin{array}{c} {\bar{V}}_{1}\\ {\bar{V}}_{2} \end{array}\right]=\left[\begin{array}{cc} \left[Z_{11}\right] & \left[Z_{12}\right]\\ \left[Z_{21}\right] & \left[Z_{22}\right] \end{array}\right]\;\left[\begin{array}{c} {\bar{I}}_{1}\\ {\bar{I}}_{2} \end{array}\right],$$ (9)

where

$$\left[Z_{11}\right]=\left[\begin{array}{ccc} Z_{ij11} & Z_{ij12} & Z_{ij13}\\ Z_{ij21} & Z_{ij22} & Z_{ij23}\\ Z_{ij31} & Z_{ij32} & Z_{ij33} \end{array}\right].$$ (10)

For each nonlinear large-signal impedance parameter $Z_{ijkl}$, the index *i* refers to the port number of the voltage *V*, the index *j* refers to the port number of the current *I*, *k* is the harmonic index of *V*, and *l* is the harmonic index of *I*. The vectors ${\bar{V}}_{i}$ and ${\bar{I}}_{j}$ are (*M*=3)-element vectors given by

$${\bar{V}}_{i}=\left[\begin{array}{c} V_{i1}\\ V_{i2}\\ V_{i3} \end{array}\right];\;{\bar{I}}_{j}=\left[\begin{array}{c} I_{i1}\\ I_{i2}\\ I_{i3} \end{array}\right]$$ (11)

Equation (9) can be expanded to

$$\left[\begin{array}{l} V_{11}\\ V_{12}\\ V_{13}\\ V_{21}\\ V_{22}\\ V_{23} \end{array}\right]=\left[\begin{array}{llllll} Z_{1111} & Z_{1112} & Z_{1113} & Z_{1211} & Z_{1212} & Z_{1213}\\ Z_{1121} & Z_{1122} & Z_{1123} & Z_{1221} & Z_{1222} & Z_{1223}\\ Z_{1131} & Z_{1132} & Z_{1133} & Z_{1231} & Z_{1232} & Z_{1233}\\ Z_{2111} & Z_{2112} & Z_{2113} & Z_{2211} & Z_{2212} & Z_{2213}\\ Z_{2121} & Z_{2122} & Z_{2123} & Z_{2221} & Z_{2222} & Z_{2223}\\ Z_{2131} & Z_{2132} & Z_{2133} & Z_{2231} & Z_{2232} & Z_{2233} \end{array}\right]\;\left[\begin{array}{l} I_{11}\\ I_{12}\\ I_{13}\\ I_{21}\\ I_{22}\\ I_{23} \end{array}\right]$$ (12)

### 2.3 Relating S and Z Matrices

The S and Z matrices can be expressed in terms of one another, if we know how *a* and *b* relate to *V* and *I*. From Eq. (1), we can express *V_ik_* in terms of *a_jl_* and *b_ik_* as follows:

$$V_{ik}=V_{ik}^{+}+V_{ik}^{-}=\sqrt{Z_{oi}}\left(a_{ik}+b_{ik}\right),$$ (13)

where the subscripts refer to the *i*th port and the *k*th harmonic. We can similarly express *I_jl_* as

$$I_{jl}=I_{jl}^{+}+I_{jl}^{-}=\frac{1}{Z_{oj}}\left(V_{jl}^{+}-V_{jl}^{-}\right)=\frac{1}{\sqrt{Z_{oj}}}\left(a_{jl}-b_{jl}\right),$$ (14)

where the subscripts refer to the *j*th port and at the *l*th harmonic.

For simplicity, we will assume for now that the network under consideration consists of two ports. Later, we can easily generalize the equations relating the S and Z matrices for any *N*-port network. If we allow the two transmission lines or waveguides connecting the two ports to have different characteristic impedances, *Z_o_*_1_ and *Z_o_*_2_, Eq. (14) can be expressed in matrix form as

$$\left[\begin{array}{c} {\bar{I}}_{1}\\ {\bar{I}}_{2} \end{array}\right]=\left[\begin{array}{cc} \left[U\right]/Z_{o1} & \left[0\right]\\ \left[0\right] & \left[U\right]/Z_{o2} \end{array}\right]\left(\left[\begin{array}{c} {\bar{V}}_{1}^{+}\\ {\bar{V}}_{2}^{+} \end{array}\right]-\left[\begin{array}{c} {\bar{V}}_{1}^{-}\\ {\bar{V}}_{2}^{-} \end{array}\right]\right),$$ (15)

where [*U*] is the identity matrix. Equation (9) can be expressed as

$$\left[\begin{array}{c} {\bar{V}}_{1}^{+}\\ {\bar{V}}_{2}^{+} \end{array}\right]+\left[\begin{array}{c} {\bar{V}}_{1}^{-}\\ {\bar{V}}_{2}^{-} \end{array}\right]=\left[\begin{array}{cc} \left[Z_{11}\right] & \left[Z_{12}\right]\\ \left[Z_{21}\right] & \left[Z_{22}\right] \end{array}\right]\;\left[\begin{array}{c} {\bar{I}}_{1}\\ {\bar{I}}_{2} \end{array}\right].$$ (16)

Combining Eqs. (15) and (16) gives

$$\begin{array}{l} \left[\begin{array}{c} {\bar{V}}_{1}^{+}\\ {\bar{V}}_{2}^{+} \end{array}\right]+\left[\begin{array}{c} {\bar{V}}_{1}^{-}\\ {\bar{V}}_{2}^{-} \end{array}\right]=\\ \left[\begin{array}{cc} \left[Z_{11}\right] & \left[Z_{12}\right]\\ \left[Z_{21}\right] & \left[Z_{22}\right] \end{array}\right]\;\left[\begin{array}{cc} \left[U\right]/Z_{o1} & \left[0\right]\\ \left[0\right] & \left[U\right]/Z_{o2} \end{array}\right]\left(\left[\begin{array}{c} {\bar{V}}_{1}^{+}\\ {\bar{V}}_{2}^{+} \end{array}\right]-\left[\begin{array}{c} {\bar{V}}_{1}^{-}\\ {\bar{V}}_{2}^{-} \end{array}\right]\right) \end{array}$$ (17)

or

$$\left[\begin{array}{c} {\bar{V}}_{1}^{+}\\ {\bar{V}}_{2}^{+} \end{array}\right]+\left[\begin{array}{c} {\bar{V}}_{1}^{-}\\ {\bar{V}}_{2}^{-} \end{array}\right]=\left[\begin{array}{cc} \left[{Z^{\prime }}_{11}\right] & \left[{Z^{\prime }}_{12}\right]\\ \left[{Z^{\prime }}_{21}\right] & \left[{Z^{\prime }}_{22}\right] \end{array}\right]\left(\left[\begin{array}{c} {\bar{V}}_{1}^{+}\\ {\bar{V}}_{2}^{+} \end{array}\right]-\left[\begin{array}{c} {\bar{V}}_{1}^{-}\\ {\bar{V}}_{2}^{-} \end{array}\right]\right),$$ (18)

where

$$\left[\begin{array}{cc} \left[{Z^{\prime }}_{11}\right] & \left[{Z^{\prime }}_{12}\right]\\ \left[{Z^{\prime }}_{21}\right] & \left[{Z^{\prime }}_{22}\right] \end{array}\right]=\left[\begin{array}{cc} \left[Z_{11}\right] & \left[Z_{12}\right]\\ \left[Z_{21}\right] & \left[Z_{22}\right] \end{array}\right]\;\left[\begin{array}{cc} \left[U\right]/Z_{o1} & \left[0\right]\\ \left[0\right] & \left[U\right]/Z_{o2} \end{array}\right]$$ (19)

is the normalized impedance matrix. Equation (18) can be rewritten as

$$\begin{array}{l} \left(\left[\begin{array}{cc} \left[{Z^{\prime }}_{11}\right] & \left[{Z^{\prime }}_{12}\right]\\ \left[{Z^{\prime }}_{21}\right] & \left[{Z^{\prime }}_{22}\right] \end{array}\right]+\left[\begin{array}{cc} \left[U\right] & \left[0\right]\\ \left[0\right] & \left[U\right] \end{array}\right]\right)\left[\begin{array}{c} {\bar{V}}_{1}^{-}\\ {\bar{V}}_{2}^{-} \end{array}\right]=\\ \left(\left[\begin{array}{cc} \left[{Z^{\prime }}_{11}\right] & \left[{Z^{\prime }}_{12}\right]\\ \left[{Z^{\prime }}_{21}\right] & \left[{Z^{\prime }}_{22}\right] \end{array}\right]-\left[\begin{array}{cc} \left[U\right] & \left[0\right]\\ \left[0\right] & \left[U\right] \end{array}\right]\right)\left[\begin{array}{c} {\bar{V}}_{1}^{+}\\ {\bar{V}}_{2}^{+} \end{array}\right] \end{array}$$ (20)

and Eq. (3) can be rewritten as

$$\begin{array}{l} \left[\begin{array}{cc} {}[U]/\sqrt{Z_{o1}} & \left[0\right]\\ \left[0\right] & [U]/\sqrt{Z_{o2}} \end{array}\right]\;[\begin{array}{c} {\bar{V}}_{1}^{-}\\ {\bar{V}}_{2}^{-} \end{array}]=\\ \left[\begin{array}{ll} \left[S_{11}\right] & \left[S_{12}\right]\\ \left[S_{21}\right] & \left[S_{22}\right] \end{array}\right]\;\left[\begin{array}{cc} {}[U]/\sqrt{Z_{o1}} & \left[0\right]\\ \left[0\right] & [U]/\sqrt{Z_{o2}} \end{array}\right]\;\left[\begin{array}{c} {\bar{V}}_{1}^{+}\\ {\bar{V}}_{2}^{+} \end{array}\right]. \end{array}$$ (21)

Combining Eqs. (20) and (21) allows us to solve for S in terms of Z:

$$\begin{array}{l} \left[\begin{array}{ll} \left[S_{11}\right] & \left[S_{12}\right]\\ \left[S_{21}\right] & \left[S_{22}\right] \end{array}\right]=\left[\begin{array}{cc} {}[U]/\sqrt{Z_{o1}} & \left[0\right]\\ \left[0\right] & [U]/\sqrt{Z_{o2}} \end{array}\right]{\left(\left[\begin{array}{ll} \left[{Z^{\prime }}_{11}\right] & \left[{Z^{\prime }}_{12}\right]\\ \left[{Z^{\prime }}_{21}\right] & \left[{Z^{\prime }}_{22}\right] \end{array}\right]+\left[\begin{array}{ll} \left[U\right] & \left[0\right]\\ \left[0\right] & \left[U\right] \end{array}\right]\right)}^{-1}\\ \left(\left[\begin{array}{ll} \left[{Z^{\prime }}_{11}\right] & \left[{Z^{\prime }}_{12}\right]\\ \left[{Z^{\prime }}_{21}\right] & \left[{Z^{\prime }}_{22}\right] \end{array}\right]-\left[\begin{array}{ll} \left[U\right] & \left[0\right]\\ \left[0\right] & \left[U\right] \end{array}\right]\right){\left[\begin{array}{cc} {}[U]/\sqrt{Z_{o1}} & \left[0\right]\\ \left[0\right] & [U]/\sqrt{Z_{o2}} \end{array}\right]}^{-1}. \end{array}$$ (22)

If *Z_o_*_1_ = *Z_o_*_2_, Eq. (22) reduces to

$$\begin{array}{r} \left[\begin{array}{cc} \left[S_{11}\right] & \left[S_{12}\right]\\ \left[S_{21}\right] & \left[S_{22}\right] \end{array}\right]={\left(\left[\begin{array}{cc} \left[{Z^{\prime }}_{11}\right] & \left[{Z^{\prime }}_{12}\right]\\ \left[{Z^{\prime }}_{21}\right] & \left[{Z^{\prime }}_{22}\right] \end{array}\right]+\left[\begin{array}{cc} \left[U\right] & \left[0\right]\\ \left[0\right] & \left[U\right] \end{array}\right]\right)}^{-1}\\ \left(\left[\begin{array}{cc} \left[{Z^{\prime }}_{11}\right] & \left[{Z^{\prime }}_{12}\right]\\ \left[{Z^{\prime }}_{21}\right] & \left[{Z^{\prime }}_{22}\right] \end{array}\right]-\left[\begin{array}{cc} \left[U\right] & \left[0\right]\\ \left[0\right] & \left[U\right] \end{array}\right]\right). \end{array}$$ (23)

Alternatively, we can combine Eqs. (20) and (21) to solve for Z in terms of S:

$$\begin{array}{c} \left[\begin{array}{ll} \left[{Z^{\prime }}_{11}\right] & \left[{Z^{\prime }}_{12}\right]\\ \left[{Z^{\prime }}_{21}\right] & \left[{Z^{\prime }}_{22}\right] \end{array}\right]=\left(\left[\begin{array}{ll} \left[U\right] & \left[0\right]\\ \left[0\right] & \left[U\right] \end{array}\right]+{\left[\begin{array}{cc} \frac{\left[U\right]}{\sqrt{Z_{o1}}} & \left[0\right]\\ \left[0\right] & \frac{\left[U\right]}{\sqrt{Z_{o2}}} \end{array}\right]}^{-1}\left[\begin{array}{ll} \left[S_{11}\right] & \left[S_{12}\right]\\ \left[S_{21}\right] & \left[S_{22}\right] \end{array}\right]\;\left[\begin{array}{cc} \frac{\left[U\right]}{\sqrt{Z_{o1}}} & \left[0\right]\\ \left[0\right] & \frac{\left[U\right]}{\sqrt{Z_{o2}}} \end{array}\right]\right)\\ {\left(\left[\begin{array}{ll} \left[U\right] & \left[0\right]\\ \left[0\right] & \left[U\right] \end{array}\right]-{\left[\begin{array}{cc} \frac{\left[U\right]}{\sqrt{Z_{o1}}} & \left[0\right]\\ \left[0\right] & \frac{\left[U\right]}{\sqrt{Z_{o2}}} \end{array}\right]}^{-1}\left[\begin{array}{ll} \left[S_{11}\right] & \left[S_{12}\right]\\ \left[S_{21}\right] & \left[S_{22}\right] \end{array}\right]\;\left[\begin{array}{cc} \frac{\left[U\right]}{\sqrt{Z_{o1}}} & \left[0\right]\\ \left[0\right] & \frac{\left[U\right]}{\sqrt{Z_{o2}}} \end{array}\right]\right)}^{-1}. \end{array}$$ (24)

If *Z_o_*_1_ = *Z_o_*_2_, Eq. (24) reduces to

$$\begin{array}{r} \left[\begin{array}{cc} \left[{Z^{\prime }}_{11}\right] & \left[{Z^{\prime }}_{12}\right]\\ \left[{Z^{\prime }}_{21}\right] & \left[{Z^{\prime }}_{22}\right] \end{array}\right]=\left(\left[\begin{array}{cc} \left[U\right] & \left[0\right]\\ \left[0\right] & \left[U\right] \end{array}\right]+\left[\begin{array}{cc} \left[S_{11}\right] & \left[S_{12}\right]\\ \left[S_{21}\right] & \left[S_{22}\right] \end{array}\right]\right)\\ {\left(\left[\begin{array}{cc} \left[U\right] & \left[0\right]\\ \left[0\right] & \left[U\right] \end{array}\right]-\left[\begin{array}{cc} \left[S_{11}\right] & \left[S_{12}\right]\\ \left[S_{21}\right] & \left[S_{22}\right] \end{array}\right]\right)}^{-1}. \end{array}$$ (25)

### 2.4 Nonlinear Large-Signal Admittance Parameters

We can also express the relationship between voltages (*V*’s) and currents (*I*’s) in terms of a nonlinear large-signal admittance matrix D, as follows

I=DV, (26)

where D is an (*N×M*)^2^-element square matrix. For a two-port network with three harmonics, for example, Eq. (26) becomes

$$\left[\begin{array}{c} {\bar{I}}_{1}\\ {\bar{I}}_{2} \end{array}\right]=\left[\begin{array}{ll} \left[D_{11}\right] & \left[D_{12}\right]\\ \left[D_{21}\right] & \left[D_{22}\right] \end{array}\right]\;\left[\begin{array}{c} {\bar{V}}_{1}\\ {\bar{V}}_{2} \end{array}\right],$$ (27)

where

$$[D_{ij}]=[\begin{array}{lll} D_{ij11} & D_{ij12} & D_{ij13}\\ D_{ij21} & D_{ij22} & D_{ij23}\\ D_{ij31} & D_{ij32} & D_{ij33} \end{array}].$$ (28)

For each nonlinear large-signal admittance parameter $D_{ijkl}$, the index *i* refers to the port number of the current *I*, the index *j* refers to the port number of the voltage *V*, *k* is the harmonic index of *I*, and *l* is the harmonic index of *V*. The vectors ${\bar{V}}_{j}$ and ${\bar{I}}_{i}$ are, once again, (*M*=3)-element vectors, defined in Eq. (11). Equation (27) can be expanded as follows

$$\left[\begin{array}{l} I_{11}\\ I_{12}\\ I_{13}\\ I_{21}\\ I_{22}\\ I_{23} \end{array}\right]=\left[\begin{array}{llllll} D_{1111} & D_{1112} & D_{1113} & D_{1211} & D_{1212} & D_{1213}\\ D_{1121} & D_{1122} & D_{1123} & D_{1221} & D_{1222} & D_{1223}\\ D_{1131} & D_{1132} & D_{1133} & D_{1231} & D_{1232} & D_{1233}\\ D_{2111} & D_{2112} & D_{2113} & D_{2211} & D_{2212} & D_{2213}\\ D_{2121} & D_{2122} & D_{2123} & D_{2221} & D_{2222} & D_{2223}\\ D_{2131} & D_{2132} & D_{2133} & D_{2231} & D_{2232} & D_{2233} \end{array}\right]\;\left[\begin{array}{l} V_{11}\\ V_{12}\\ V_{13}\\ V_{21}\\ V_{22}\\ V_{23} \end{array}\right].$$ (29)

### 2.5 Relating S and D Matrices

The S and D matrices can also be expressed in terms of one another, using Eqs. (13) and (14) which show how *a* and *b* relate to *V* and *I*.

For simplicity, we will again assume the network under consideration consists of two ports. If we allow the two transmission lines or waveguides connecting the two ports to have different characteristic impedances *Z_o_*_1_ and *Z_o_*_2_, Eq. (14) can be expressed in matrix form as

$$\left[\begin{array}{c} {\bar{I}}_{1}\\ {\bar{I}}_{2} \end{array}\right]=\left[\begin{array}{cc} {}[U]/Z_{o1} & \left[0\right]\\ \left[0\right] & [U]/Z_{o2} \end{array}\right]\left(\left[\begin{array}{c} {\bar{V}}_{1}^{+}\\ {\bar{V}}_{2}^{+} \end{array}\right]-\left[\begin{array}{c} {\bar{V}}_{1}^{-}\\ {\bar{V}}_{2}^{-} \end{array}\right]\right),$$ (30)

where [*U*] is the identity matrix. Equation (27) can be expressed as

$$\left[\begin{array}{c} {\bar{I}}_{1}\\ {\bar{I}}_{2} \end{array}\right]=\left[\begin{array}{ll} \left[D_{11}\right] & \left[D_{12}\right]\\ \left[D_{21}\right] & \left[D_{22}\right] \end{array}\right]\left(\left[\begin{array}{c} {\bar{V}}_{1}^{+}\\ {\bar{V}}_{2}^{+} \end{array}\right]+\left[\begin{array}{c} {\bar{V}}_{1}^{-}\\ {\bar{V}}_{2}^{-} \end{array}\right]\right).$$ (31)

Combining Eqs. (30) and (31) gives

$$\left[\begin{array}{c} {\bar{V}}_{1}^{+}\\ {\bar{V}}_{2}^{+} \end{array}\right]-\left[\begin{array}{c} {\bar{V}}_{1}^{-}\\ {\bar{V}}_{2}^{-} \end{array}\right]={\left[\begin{array}{cc} {}[U]/Z_{o1} & \left[0\right]\\ \left[0\right] & [U]/Z_{o2} \end{array}\right]}^{-1}\left[\begin{array}{ll} \left[D_{11}\right] & \left[D_{12}\right]\\ \left[D_{21}\right] & \left[D_{22}\right] \end{array}\right]\left(\left[\begin{array}{c} {\bar{V}}_{1}^{+}\\ {\bar{V}}_{2}^{+} \end{array}\right]+\left[\begin{array}{c} {\bar{V}}_{1}^{-}\\ {\bar{V}}_{2}^{-} \end{array}\right]\right)$$ (32)

or

$$\left[\begin{array}{c} {\bar{V}}_{1}^{+}\\ {\bar{V}}_{2}^{+} \end{array}\right]-\left[\begin{array}{c} {\bar{V}}_{1}^{-}\\ {\bar{V}}_{2}^{-} \end{array}\right]=\left[\begin{array}{ll} \left[{D^{\prime }}_{11}\right] & \left[{D^{\prime }}_{12}\right]\\ \left[{D^{\prime }}_{21}\right] & \left[{D^{\prime }}_{22}\right] \end{array}\right]\left(\left[\begin{array}{c} {\bar{V}}_{1}^{+}\\ {\bar{V}}_{2}^{+} \end{array}\right]+\left[\begin{array}{c} {\bar{V}}_{1}^{-}\\ {\bar{V}}_{2}^{-} \end{array}\right]\right),$$ (33)

where

$$\left[\begin{array}{ll} \left[{D^{\prime }}_{11}\right] & \left[{D^{\prime }}_{12}\right]\\ \left[{D^{\prime }}_{21}\right] & \left[{D^{\prime }}_{22}\right] \end{array}\right]={\left[\begin{array}{cc} {}[U]/Z_{o1} & \left[0\right]\\ \left[0\right] & [U]/Z_{o2} \end{array}\right]}^{-1}\left[\begin{array}{ll} \left[D_{11}\right] & \left[D_{12}\right]\\ \left[D_{21}\right] & \left[D_{22}\right] \end{array}\right]$$ (34)

is the normalized admittance matrix. Equation (33) can be rewritten as

$$\left(\left[\begin{array}{ll} \left[U\right] & \left[0\right]\\ \left[0\right] & \left[U\right] \end{array}\right]+\left[\begin{array}{ll} \left[{D^{\prime }}_{11}\right] & \left[{D^{\prime }}_{12}\right]\\ \left[{D^{\prime }}_{21}\right] & \left[{D^{\prime }}_{22}\right] \end{array}\right]\right)\left[\begin{array}{c} {\bar{V}}_{1}^{-}\\ {\bar{V}}_{2}^{-} \end{array}\right]=\left(\left[\begin{array}{ll} \left[U\right] & \left[0\right]\\ \left[0\right] & \left[U\right] \end{array}\right]-\left[\begin{array}{ll} \left[{D^{\prime }}_{11}\right] & \left[{D^{\prime }}_{12}\right]\\ \left[{D^{\prime }}_{21}\right] & \left[{D^{\prime }}_{22}\right] \end{array}\right]\right)\left[\begin{array}{c} {\bar{V}}_{1}^{+}\\ {\bar{V}}_{2}^{+} \end{array}\right]$$ (35)

and Eq. (3) can be rewritten as

$$\left[\begin{array}{cc} {}[U]/\sqrt{Z_{o1}} & \left[0\right]\\ \left[0\right] & [U]/\sqrt{Z_{o1}} \end{array}\right]\;\left[\begin{array}{c} {\bar{V}}_{1}^{-}\\ {\bar{V}}_{2}^{-} \end{array}\right]=\left[\begin{array}{ll} \left[S_{11}\right] & \left[S_{12}\right]\\ \left[S_{21}\right] & \left[S_{22}\right] \end{array}\right]\;\left[\begin{array}{cc} {}[U]/\sqrt{Z_{o1}} & \left[0\right]\\ \left[0\right] & [U]/\sqrt{Z_{o2}} \end{array}\right]\;\left[\begin{array}{c} {\bar{V}}_{1}^{+}\\ {\bar{V}}_{2}^{+} \end{array}\right]$$ (36)

Combining Eqs. (35) and (36) allows us to solve for S in terms of D:

$$\begin{array}{c} \left[\begin{array}{ll} \left[S_{11}\right] & \left[S_{12}\right]\\ \left[S_{21}\right] & \left[S_{22}\right] \end{array}\right]=\left[\begin{array}{cc} {}[U]/\sqrt{Z_{o1}} & \left[0\right]\\ \left[0\right] & [U]/\sqrt{Z_{o2}} \end{array}\right]{\left(\left[\begin{array}{ll} \left[U\right] & \left[0\right]\\ \left[0\right] & \left[U\right] \end{array}\right]+\left[\begin{array}{ll} \left[{D^{\prime }}_{11}\right] & \left[{D^{\prime }}_{12}\right]\\ \left[{D^{\prime }}_{21}\right] & \left[{D^{\prime }}_{22}\right] \end{array}\right]\right)}^{-1}\\ \left(\left[\begin{array}{ll} \left[U\right] & \left[0\right]\\ \left[0\right] & \left[U\right] \end{array}\right]-\left[\begin{array}{ll} \left[{D^{\prime }}_{11}\right] & \left[{D^{\prime }}_{12}\right]\\ \left[{D^{\prime }}_{21}\right] & \left[{D^{\prime }}_{22}\right] \end{array}\right]\right){\left[\begin{array}{cc} {}[U]/\sqrt{Z_{o1}} & \left[0\right]\\ \left[0\right] & [U]/\sqrt{Z_{o2}} \end{array}\right]}^{-1}. \end{array}$$ (37)

If *Z_o_*_1_ = *Z_o_*_2_, Eq. (37) reduces to:

$$\begin{array}{r} \left[\begin{array}{cc} \left[S_{11}\right] & \left[S_{12}\right]\\ \left[S_{21}\right] & \left[S_{22}\right] \end{array}\right]={\left(\left[\begin{array}{cc} \left[U\right] & \left[0\right]\\ \left[0\right] & \left[U\right] \end{array}\right]+\left[\begin{array}{cc} \left[{D^{\prime }}_{11}\right] & \left[{D^{\prime }}_{12}\right]\\ \left[{D^{\prime }}_{21}\right] & \left[{D^{\prime }}_{22}\right] \end{array}\right]\right)}^{-1}\\ \left(\left[\begin{array}{cc} \left[U\right] & \left[0\right]\\ \left[0\right] & \left[U\right] \end{array}\right]-\left[\begin{array}{cc} \left[{D^{\prime }}_{11}\right] & \left[{D^{\prime }}_{12}\right]\\ \left[{D^{\prime }}_{21}\right] & \left[{D^{\prime }}_{22}\right] \end{array}\right]\right) \end{array}$$ (38)

Alternatively, we can combine Eqs. (35) and (36) to solve for D in terms of S:

$$\begin{array}{c} \left[\begin{array}{ll} \left[{D^{\prime }}_{11}\right] & \left[{D^{\prime }}_{12}\right]\\ \left[{D^{\prime }}_{21}\right] & \left[{D^{\prime }}_{22}\right] \end{array}\right]=\left(\left[\begin{array}{ll} \left[U\right] & \left[0\right]\\ \left[0\right] & \left[U\right] \end{array}\right]-{\left[\begin{array}{ll} \frac{\left[U\right]}{\sqrt{Z_{o1}}} & \left[0\right]\\ \left[0\right] & \frac{\left[U\right]}{\sqrt{Z_{o2}}} \end{array}\right]}^{-1}\left[\begin{array}{ll} \left[S_{11}\right] & \left[S_{12}\right]\\ \left[S_{21}\right] & \left[S_{22}\right] \end{array}\right]\;\left[\begin{array}{ll} \frac{\left[U\right]}{\sqrt{Z_{o1}}} & \left[0\right]\\ \left[0\right] & \frac{\left[U\right]}{\sqrt{Z_{o2}}} \end{array}\right]\right)\\ {\left(\left[\begin{array}{ll} \left[U\right] & \left[0\right]\\ \left[0\right] & \left[U\right] \end{array}\right]+{\left[\begin{array}{ll} \frac{\left[U\right]}{\sqrt{Z_{o1}}} & \left[0\right]\\ \left[0\right] & \frac{\left[U\right]}{\sqrt{Z_{o2}}} \end{array}\right]}^{-1}\left[\begin{array}{ll} \left[S_{11}\right] & \left[S_{12}\right]\\ \left[S_{21}\right] & \left[S_{22}\right] \end{array}\right]\;\left[\begin{array}{ll} \frac{\left[U\right]}{\sqrt{Z_{o1}}} & \left[0\right]\\ \left[0\right] & \frac{\left[U\right]}{\sqrt{Z_{o2}}} \end{array}\right]\right)}^{-1} \end{array}$$ (39)

If *Z_o_*_1_ = *Z_o_*_2_, Eq. (39) reduces to

$$\begin{array}{r} \left[\begin{array}{cc} \left[{D^{\prime }}_{11}\right] & \left[{D^{\prime }}_{12}\right]\\ \left[{D^{\prime }}_{21}\right] & \left[{D^{\prime }}_{22}\right] \end{array}\right]=\left(\left[\begin{array}{cc} \left[U\right] & \left[0\right]\\ \left[0\right] & \left[U\right] \end{array}\right]-\left[\begin{array}{cc} \left[S_{11}\right] & \left[S_{12}\right]\\ \left[S_{21}\right] & \left[S_{22}\right] \end{array}\right]\right)\\ {\left(\left[\begin{array}{cc} \left[U\right] & \left[0\right]\\ \left[0\right] & \left[U\right] \end{array}\right]+\left[\begin{array}{cc} \left[S_{11}\right] & \left[S_{12}\right]\\ \left[S_{21}\right] & \left[S_{22}\right] \end{array}\right]\right)}^{-1} \end{array}$$ (40)

### 2.6 One-Port Network With Single-Tone Excitation

For a one-port network with a single-tone excitation at the fundamental frequency, we can extract a reflection coefficient given by

$${S_{11k1}=\frac{\left|b_{1k}|∠(\phi_{b_{1k}}-k\phi_{a_{11}}\right)}{\left|a_{11}\right|}|}_{a_{1m}=0\;\text{for all}\;m(m\neq 1)}.$$ (41)

The limitation imposed on the equation is that all other incident waves other than *a*_11_equal zero. Instead of simply taking the ratio of *b*_1_*_k_* to *a*_11_, we reference the phase of *b*_1_*_k_* to that of *a*_11_. To do this, we must subtract *k* times the phase of *a*_11_ from *b*_1_*_k_* [8].

For a one-port network with a single-tone excitation at the fundamental frequency, we can show that the equation relating S and Z reduces to the same well-known equation for the linear case if we assume that no energy is redistributed into the form of frequency down-conversion. To illustrate this, we will consider only *M*=3 harmonics, for the sake of simplicity. Equation (6) reduces to

$$\left[\begin{array}{c} b_{11}\\ b_{12}\\ b_{13} \end{array}\right]=\left[\begin{array}{lll} S_{1111} & S_{1112} & S_{1113}\\ S_{1121} & S_{1122} & S_{1123}\\ S_{1131} & S_{1132} & S_{1133} \end{array}\right]\;\left[\begin{array}{c} a_{11}\\ 0\\ 0 \end{array}\right],$$ (42)

for a one-port network with a single-tone excitation *a*_11_. This matrix can be rewritten as a set of three equations:

$$b_{11}=S_{1111}a_{11};\;b_{12}=S_{1121}a_{11};\;b_{13}=S_{1131}a_{11}.$$ (43)

Likewise, Eq. (12) reduces to

$$\left[\begin{array}{c} V_{11}\\ V_{12}\\ V_{13} \end{array}\right]=\left[\begin{array}{lll} Z_{1111} & Z_{1112} & Z_{1113}\\ Z_{1121} & Z_{1122} & Z_{1123}\\ Z_{1131} & Z_{1132} & Z_{1133} \end{array}\right]\;\left[\begin{array}{c} I_{11}\\ I_{12}\\ I_{13} \end{array}\right],$$ (44)

where the voltage *V*_11_ at the first harmonic can be expressed as

$$V_{11}=Z_{1111}I_{11}+Z_{1112}I_{12}+Z_{1113}I_{13}.$$ (45)

From Eqs. (13) and (14), we know that

$$\begin{array}{l} V_{11}=\sqrt{Z_{o1}}(a_{11}+b_{11}),\\ I_{11}=\frac{1}{\sqrt{Z_{o1}}}(a_{11}-b_{11}),\\ I_{12}=\frac{1}{\sqrt{Z_{o1}}}(a_{12}-b_{12})=-\frac{b_{12}}{\sqrt{Z_{o1}}},\\ I_{13}=\frac{1}{\sqrt{Z_{o1}}}(a_{13}-b_{13})=-\frac{b_{13}}{\sqrt{Z_{o1}}}. \end{array}$$ (46)

Combining Eqs. (45) and (46) gives

$$\sqrt{Z_{o1}}(a_{11}+b_{11})=\frac{1}{\sqrt{Z_{o1}}}[Z_{1111}(a_{11}-b_{11})-Z_{1112}b_{12}-Z_{1112}b_{13}].$$ (47)

Substituting Eq. (43) into Eq. (47) and solving for $Z_{1111}$ gives

$$Z_{1111}=\frac{Z_{o1}(1+S_{1111})+Z_{1112}S_{1121}+Z_{1113}S_{1131}}{\left(1-S_{1111}\right)}.$$ (48)

If no energy is redistributed into the form of frequency down-conversion (i.e., $Z_{1112}\;=\;Z_{1113\;}\;=\;0$), then Eq. (48) reduces to the same equation as in the linear case:

$$Z_{11}=Z_{o1}\frac{\left(1+S_{11}\right)}{\left(1-S_{11}\right)}.$$ (49)

A similar derivation can be performed to show that

$$D_{1111}=\frac{(1-S_{1111})/Z_{o1}-D_{1112}S_{1121}-D_{1113}S_{1131}}{\left(1+S_{1111}\right)}.$$ (50)

Once again, if no energy is transferred to frequency down-conversion (i.e., $D_{1112}\;=\;D_{1113}\;=\;0$), then Eq. (50) reduces to the same equation as in the linear case:

$$Y_{11}=\frac{1}{Z_{11}}=\frac{1}{Z_{o1}}\frac{\left(1-S_{11}\right)}{\left(1+S_{11}\right)}.$$ (51)

### 2.7 Two-Port Network With Single-Tone Excitation

For a two-port network excited at port 1 by a single-tone excitation at the fundamental frequency, we can extract an input reflection coefficient given by

$${S_{11k1}=\frac{\left|b_{1k}|∠(\phi_{b_{1k}}-k\phi_{a_{11}}\right)}{\left|a_{11}\right|}|}_{a_{mm}=0\;\text{for all}\;m,n[(m\neq 1)\wedge (n\neq 1)]}.$$ (52)

As with Eq. (41), instead of simply taking the ratio of *b*_1_*_k_* to *a*_11_, we phase reference to *a*_11_. To do this we must subtract *k* times the phase of *a*_11_ from *b*_1_*_k_*. The limitation once again imposed on the equation is that all other incident waves other than *a*_11_ equal zero.

Another valuable parameter, the forward transmission coefficient, is similarly extracted as follows

$${S_{21k1}=\frac{\left|b_{2k}|∠(\phi_{b_{2k}}-k\phi_{b_{11}}\right)}{\left|a_{11}\right|}|}_{a_{mm}=0\;\text{for all}\;m,n[(m\neq 1)\wedge (n\neq 1)]}.$$ (53)

This parameter provides a value of the gain or loss through a device either at the fundamental frequency or converted to a higher harmonic frequency.

In addition to the previous two parameters, given in Eqs. (52) and (53), an output reflection coefficient can also be useful when trying to determine the output matching network. If a nonlinear DUT is operating under its normal drive condition (*a*_11_ at some constant signal level), and a second source, excited by a small-signal tone at frequency *f_k_*, is placed at port 2 of the DUT, one of the equations in the matrix defined by Eq. (6) reduces to

$$b_{2k}=S_{21k1}a_{11}+S_{22kk}a_{2k}.$$ (54)

If we solve Eq. (54) for $S_{22kk}$, we obtain

$$S_{22kk}=\frac{b_{2k}}{a_{2k}}-\frac{S_{21k1}a_{11}}{a_{2k}}.$$ (55)

In Eq. (55), the output reflection coefficient $S_{22kk}$ obviously cannot be determined by simply taking the ratio of *b*_2_*_k_* to *a*_2_*_k_*, since the ratio also depends on *a*_11_ through $S_{21k1}$. When *a*_2_*_k_* is small, we can generate another signal ∆*a*_2_*_k_* that is offset slightly from the frequency of interest *f_k_* by ∆*f_k_*. Eq. (54) then becomes

$$b_{2k}+\Delta b_{2k}=S_{21k1}a_{11}+S_{22kk}(a_{2k}+\Delta a_{2k}),$$ (56)

where ∆*a*_2_*_k_* << *a*_2_*_k_* and $S_{22kk}$ remains constant over this frequency range. Subtracting Eq. (54) from Eq. (56) gives

$$\Delta b_{2k}=S_{22kk}\Delta a_{2k},$$ (57)

which does not depend on $S_{21k1}$. If we solve Eq. (57) for $S_{22kk}$, we obtain

$$S_{22kk}={\frac{\Delta b_{2k}}{\Delta a_{2k}}|}_{\text{Large}\;a_{11},\;\text{Small}\;\Delta a_{2k}}.$$ (58)

Equation (58) is a quasi-linear approximation of the output reflection coefficient under normal operating conditions, and is consistent with the definition of “Hot *S*_22_,” which has been used to measure the degree of mismatch at the output port of a power amplifier at its excitation frequency.

### 2.8 Summary of Sec. 2

In this section, we presented the general form of nonlinear large-signal S-parameters. Unlike linear *S*-parameters, nonlinear large-signal S-parameters depend upon the signal magnitude and must take into account the harmonic content of the input and output signals, since energy can be transferred to other frequencies in a nonlinear device. We also introduced nonlinear large-signal impedance ( Z) and admittance ( D) parameters, and presented equations for relating the different representations. Next, we made two simplifications, considering the cases of a one-port network with a single-tone excitation and a two-port network with a single-tone excitation. For the one-port case with a single-tone excitation at the fundamental frequency, we showed that the equation relating S and Z reduces to the same well-known equation for the linear case if we assume that no energy is transferred to frequency down-conversion. For the two-port case excited at port 1 by a single-tone excitation at the fundamental frequency, we extracted an input reflection coefficient $S_{11k1}$, a forward transmission coefficient $S_{21k1}$, and a quasi-linear output reflection coefficient $S_{22kk}$.

## 3. Using Nonlinear Large-Signal S-Parameters to Design a Diode Frequency-Doubler Circuit With a Harmonic-Balance Simulator

**Fig. 1:**
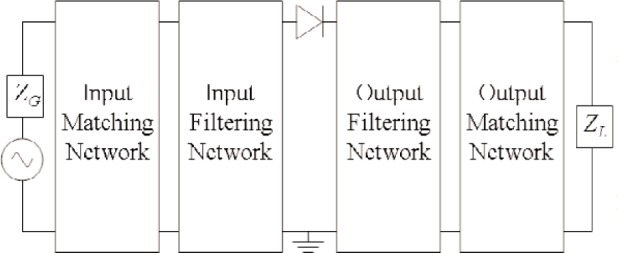
Block diagram of a single-diode resistive doubler.

Resistive frequency doublers operate on the principle that a sinusoidal waveform is distorted by the nonlinear *I*/*V* characteristic of a Schottky-barrier diode [9]. This distortion causes power to be generated at higher-harmonic frequencies. The design of such doublers involves separating the input and output signals by filters and determining the optimum input and output matching circuits, as illustrated in Fig. 1.

Although single-diode resistive doublers are not very efficient (analysis predicts a conversion loss of at least 9 dB [10]), we chose this circuit because it is simple enough to clearly illustrate how nonlinear large-signal S-parameters can be used as a design tool.

**Fig. 2:**
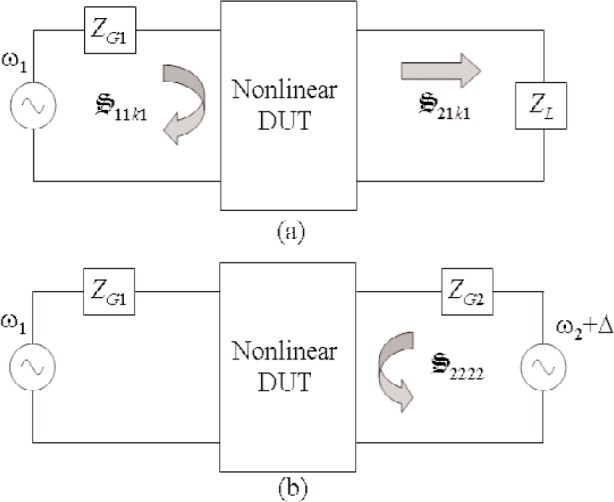
Nonlinear large-signal S-parameters used to characterize a two-port device excited by a single-tone signal at port 1.

In the following sections, we describe the various steps involved in designing a single-diode 1 GHz frequency-doubler circuit. Since we are using a simulator, we can force the stimulus to consist of only |*a*_11_|, with all other *a_mn_* terms equal to zero, where *m* and *n* are positive integers such that *m* ≠ 1 and *n* ≠ 1. (In practice, this condition can never be completely realized in a measurement environment.) With only an *a*_11_ component present, we need only consider the parameters $S_{11k1}$ (Eq. 52), which is a measure of the large-signal input match at the *k*th harmonic, as well as the parameter $S_{21k1}$ (Eq. 53), a measure of the large-signal conversion loss or gain at the *k*th harmonic, plus the quasilinear $S_{2222}$ (Eq. 58) to determine the output matching network at the second harmonic. Figure 2 illustrates the setups required for determining these parameters. Determining $S_{2222}$ requires a second source at port 2 at a frequency slightly offset from *ω*_2_.

In the first step, we perform a simulation on the diode alone and use $S_{2121}$ to determine the optimum bias condition for converting power from the fundamental frequency to the second harmonic. Second, we add filtering networks to separate the input and output signals, and verify their proper performance by looking at $S_{2111}$ and $S_{1121}$. Third, we make use of $S_{1111}$ to determine the input matching network. Fourth, with the input matching network in place, we place a second source at port 2 and find the quasi-linear value of $S_{2222}$, which allows us to determine the output matching network. Fifth, we use the optimization feature of the simulator to minimize $S_{1111}$ by varying the line lengths of the input and output matching circuits. And finally, sixth, we add 4 GHz and 6 GHz filters at the output (and re-determine the proper input and output matching circuits) in order to reduce the values of $S_{2141}$ and $S_{2161}$, which in turn increases the value of $S_{2121}$ and cleans up the output waveform.

### 3.1 Diode Only

In this example, we use a compact model to simulate a commercial Schottky-barrier diode. The model includes a series resistance *R*_s_ of 14 Ω, a junction capacitance at zero voltage *C_j_*_0_ of 0.08 pF, and a reverse saturation current *I*_s_ of 3 ×10^−10^ A.

First, we perform a harmonic-balance simulation on the diode, sweeping the bias voltage to determine which condition gives the highest value of $S_{2121}$ for *a*_11_ = 1.0 V. Note that in all simulations we set the generator impedance *Z*_G_ and the load impedance *Z*_L_ to 50 Ω After sweeping the voltage, we determine that the optimum forward bias is +0.48 V.

### 3.2 Diode With 1 GHz and 2 GHz Filters

With a stimulus of *a*_11_ = 1.0 V and a forward bias of +0.48 V, we add filtering networks to separate the input and output signals. On the input side, we place a 2 GHz, *λ*/4 (*λ*/8 at 1 GHz) open-circuited stub. This creates an RF short at 2 GHz, preventing the output power generated in the diode from traveling backward. On the output side, we place a 1 GHz, *λ*/4 open-circuited stub. This creates an RF short at 1 GHz, preventing any signal at 1 GHz from traveling forward.

Table 1 lists the simulated values for $S_{1111}-S_{1161},\;S_{2111}-S_{2161},\;G_{2}$ and $G_{2}/G$ for each of the design stages, where G is the expanded power gain and $G_{2}$ is the expanded power gain confined to the second harmonic, as defined in [11]. With the 1 GHz and 2 GHz filters in place, we see that the value of $\left|S_{1121}\right|$ decreases from 0.170 to 1.3 × 10^−5^, the value of $\left|S_{2111}\right|$ decreases from 0.536 to 3.3 × 10^−5^, and $G_{2}$ increases from −14.16 dB to −9.73 dB.

### 3.3 Diode With 1 GHz and 2 GHz Filters and Input Matching

Once the filters are placed in the circuit, we make use of the complex-valued $S_{1111}$ to design the input matching network with the well-known single, open-circuited stub technique. This is possible, assuming that no energy is transferred to frequency down-conversion, as discussed in Sec. 2.6. We see in Table 1 that $\left|S_{1111}\right|$ reduces from 0.569 without the input matching network to 9.4 × 10^−2^ with the input matching network in place. Likewise, $G_{2}$ increases from −9.73 dB to −9.69 dB.

### 3.4 Diode With 1 GHz and 2 GHz Filters, Plus Input and Output Matching

Whereas our input matching network is designed for 1 GHz, our output matching network must be designed for 2 GHz. While the circuit is operating under its normal drive condition (*a*_11_ = 1.0 V and a forward bias of +0.48 V) we place a second source at port 2, excited by a small-signal tone (Δ*a*_22_ = 0.01 V) at a frequency offset of 10 kHz from the desired 2 GHz, to give us the quasi-linear value of $S_{2222}$, which allows us to determine the output matching network. We make use of $S_{2222}$ to design the output matching network with the well-known single, open-circuited stub technique. We see in Table 1 that with the output matching network in place, the value of $\left|S_{2121}\right|$ is only marginally increased from 0.326 to 0.328. This is because the value of $S_{2222}$ is relatively low, which means the output is already almost matched to 50 Ω. We also note that $G_{2}$ increases from −9.69 dB to −9.65 dB.

### 3.5 Diode With 1 GHz and 2 GHz Filters, Plus Optimized Input and Output Matching

With the filters and matching networks in place, we use the optimization feature of the simulator to minimize $S_{1111}$by varying the lengths of the lines in the input and output matching circuits. Doing this decreases the value of $\left|S_{1111}\right|$ from 8.7 × 10^−2^ to 6.0 × 10^−3^ while increasing the value of $\left|S_{2121}\right|$ from 0.328 to 0.331 and $G_{2}$ from −9.65 dB to −9.60 dB.

### 3.6 Diode With (1, 2, 4, and 6) GHz Filters, Plus Optimized Input and Output Matching

From Table 1, we see that at the output port, $\left|S_{2111}\right|,\;\left|S_{2131}\right|$, and $\left|S_{2151}\right|$ all have values less than or equal to 4.0 × 10^−5^, but $\left|S_{2141}\right|$ and $\left|S_{2161}\right|$ have noticeably higher values (at least 2.9 × 10^−2^).

**Fig. 3:**
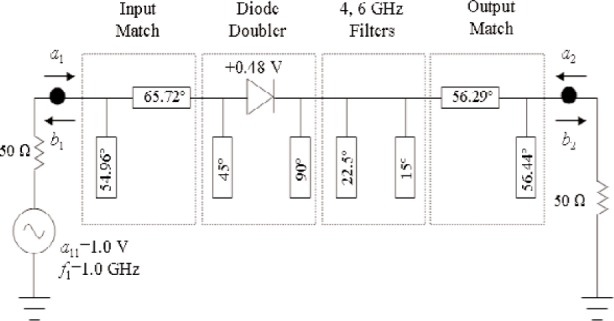
Final design of the single-diode resistive frequency doubler. Electrical lengths shown are all at 1 GHz.

**Fig. 4:**
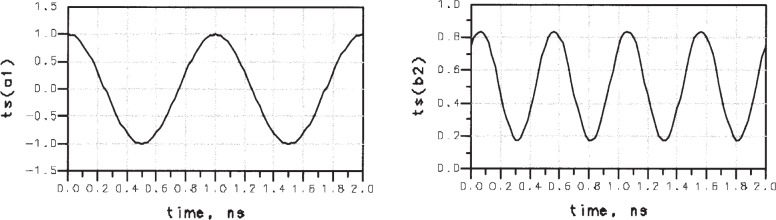
Time-domain plots of *a*_1_ and *b*_2_ for the simulated 1 GHz frequency-doubler circuit.

In order to clean up the output waveform, we add 4 GHz and 6 GHz filters, in the form of *λ*/4 open-circuited stubs, at the output. With these filters placed in the circuit, we re-determine the proper input and output matching conditions. After optimizing the circuit once again, the value of $\left|S_{2141}\right|$ decreases from 4.0 × 10^−2^ to 1.4 × 10^−6^ and the value of $\left|S_{2161}\right|$ decreases from 2.9 ×10^−2^ to 2.7 × 10^−6^. The addition of these filters, in turn, slightly increases $\left|S_{2121}\right|$ from 0.331 to 0.332 and $G_{2}$ from −9.60 dB to −9.56 dB. At this final design stage, the overall power gain is nearly −9.56 dB since the ratio $G_{2}/G=0.999$. The semi-empirical analysis of [10] predicts a maximum gain of −9 dB. Figure 3 illustrates the final design of the single-diode resistive doubler circuit. And Fig. 4 shows the time-domain plots of *a*_1_ and *b*_2_ for the final design of the simulated 1 GHz frequency-doubler circuit.

### 3.7 Summary of Sec. 3

We illustrated how nonlinear large-signal S-parameters can be used as a tool in the design process of a single-diode 1 GHz frequency-doubler. Specifically, we used $S_{1111}$ to determine the input matching network, $S_{2222}$ to determine the output matching network, and $S_{11k1},S_{21k1}$ (for *k* = 1 to 6), and $G_{2}$ to quantify the performance of the circuit at each stage.

By the final stage of the design, we had created a doubler with an overall power gain of −9.56 dB, not far from the maximum possible predicted value of −9 dB.

## 4. Determining Nonlinear Large-Signal S-Parameters from Artificial Neural Network Models Trained With Measurement Data

**Fig. 5:**
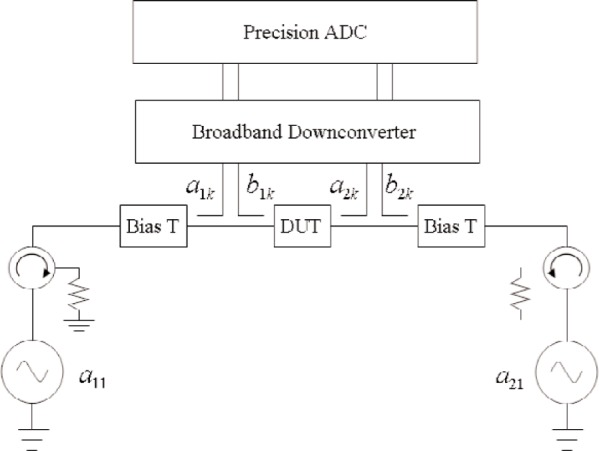
Block diagram of a nonlinear vector network analyzer equipped with a second source and isolators.

Although nonlinear large-signal S-parameters can be easily determined for an existing model in a commercial harmonic balance simulator by forcing all *a*’s other than *a*_11_ to zero, they cannot be determined directly from measurements. With currently available NVNAs, the nonlinear DUT, in conjunction with the impedance mismatches and harmonics from the system make it impossible to set all *a*’s other than *a*_11_ (assuming port 1 excitation) to zero. In order to overcome this obstacle, we propose a method [12] that makes use of multiple measurements of a DUT using a second source with isolators, as shown in Fig. 5. This measurement set-up is similar to that introduced by Verspecht et al. [6–7] to generate “nonlinear scattering functions.” As a side note, we compare and contrast the “nonlinear scattering functions” with our definitions of nonlinear large-signal scattering parameters in the Appendix.

### 4.1 Methodology

**Fig. 6:**
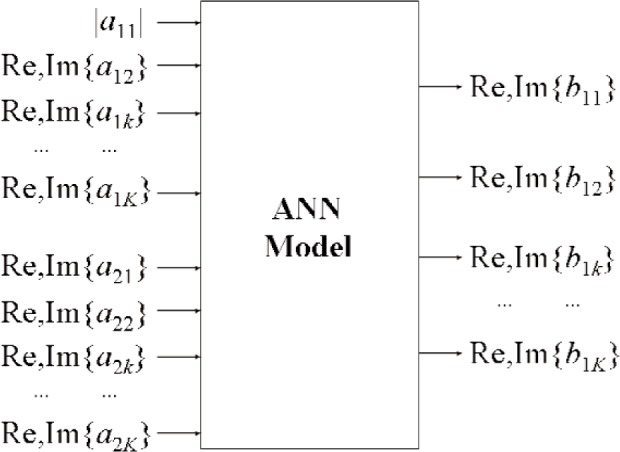
An ANN model that maps real and imaginary values of *a*’s to *b*’s for different real and imaginary values of *a_mn_* [(*m*≠1)∧(*n*≠1)]

**Fig. 7:**
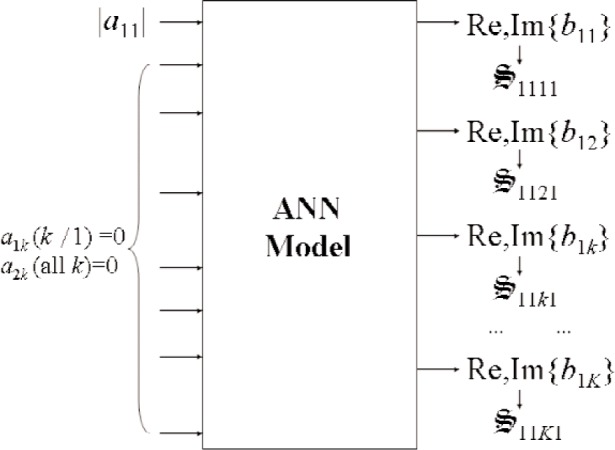
An ANN model that interpolates *b*’s from the measured results for nonzero values of *a_mn_* [(*m*≠1)∧(*n*≠1)] to the desired values for *a_mn_* [(*m*≠1)∧(*n*≠1)] equal to zero. Outputs of the ANN model yield values of $S_{11k1}$.

To illustrate our technique of generating nonlinear large-signal S-parameters, let us consider the case where a DUT is excited at port 1 by a single-tone signal at frequency *f*_1_ and signal level |*a*_11_|. Utilizing a second source, we take multiple measurements of a nonlinear circuit for different values of *a_mn_* [(*m*≠1)∧(*n*≠1)]. We then use these data to develop an artificial neural network (ANN) model that maps values of *a*’s to *b*’s, as shown in Fig. 6. Once the ANN model is trained and verified, the nonlinear large-signal S-parameters are obtained by interpolating *b*’s from the measured results for nonzero values of *a_mn_* [(*m*≠1)∧(*n*≠1)] to the desired values for *a_mn_* [(*m*≠1)∧(*n*≠1)] equal to zero, as shown in Fig. 7. Alternatively, other conditions may be called for, where $a_{mn}\neq 0$ depending on the desired application-specific figure of merit.

One popular type of ANN architecture, which is used in our work, is a feed-forward, three-layer perceptron structure (MLP3) consisting of an input layer, a hidden layer, and an output layer [13]. The hidden layer allows for complex models of input-output relationships. ANNs learn relationships among sets of input-output data that are characteristic of the device or system under consideration. After the input vectors are presented to the input neurons and output vectors are computed, the ANN outputs are compared to the desired outputs and errors are calculated. Error derivatives are then calculated and summed for each weight until all of the training sets have been presented to the network. The error derivatives are used to update the weights for the neurons, and training continues until the errors become no greater than prescribed values. In our study, we have utilized software developed by Zhang et al. [14] to construct our ANN models.

**Fig. 8:**
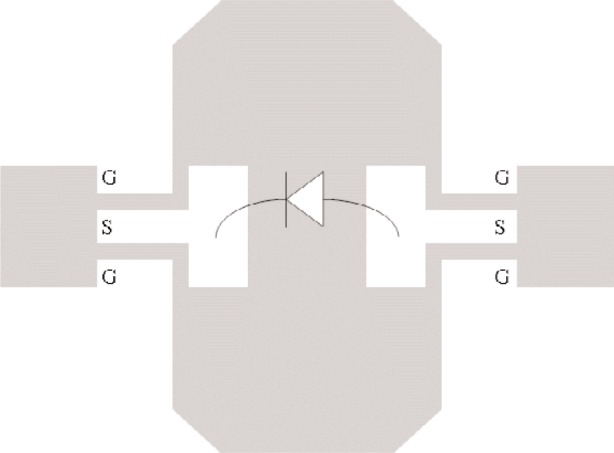
Schottky diode in a series configuration located in the middle of a CPW transmission line. (White area is metal.)

To test our method of generating nonlinear large-signal S-parameters, we fabricated a wafer-level test circuit using a Schottky diode in a series configuration, as shown in Fig. 8. The two-port diode circuit was fabricated on an alumina substrate by bonding a beam-lead diode package to the gold metalization layer with silver epoxy. The diode was located in the middle of the coplanar waveguide (CPW) transmission lines, with short lines connecting the diode to probe pads at both ports. We measured the test circuit on an NVNA using an on-wafer VNA line-reflect-reflect-match (LRRM) calibration, along with signal amplitude and phase calibrations. This process places the reference plane at the tips of the wafer probes used to connect with the CPW leads.

**Fig. 9:**
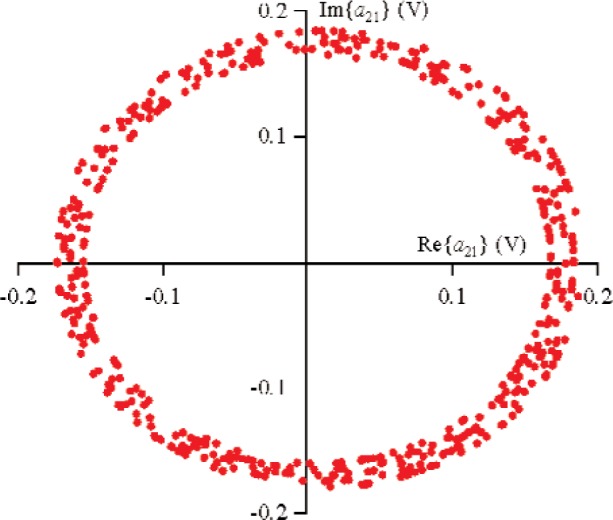
Five hundred measurements of *a*_21_in the complex plane with the excitation from source 1 held constant and the output from source 2 set to random phases with constant amplitude.

**Fig. 10:**
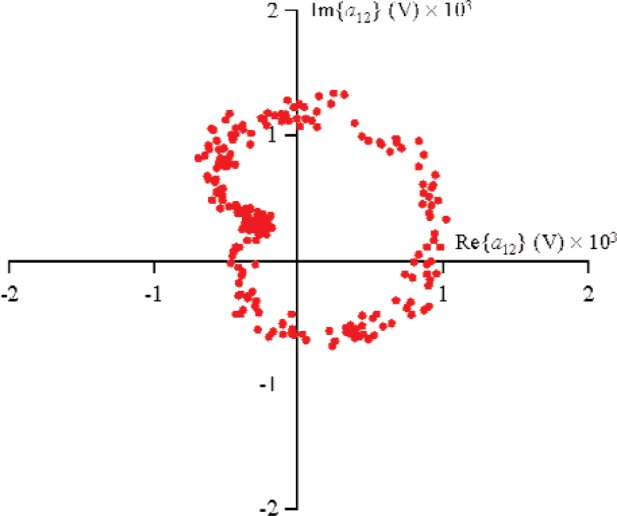
Five hundred measurements of *a*_12_ in the complex plane with the excitation from source 1 held constant and the output from source 2 set to random phases with constant amplitude.

**Fig. 11:**
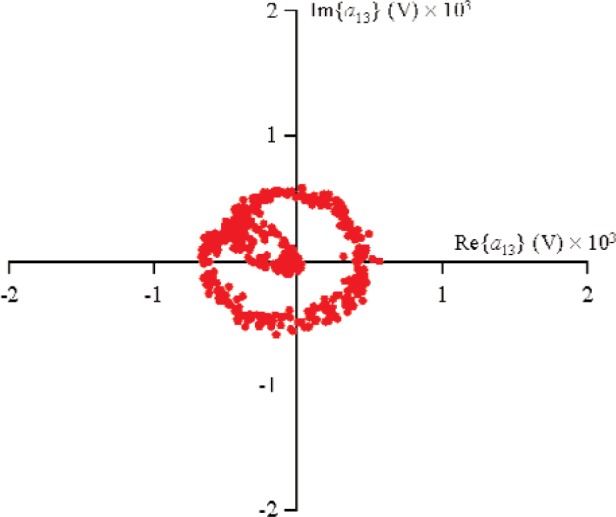
Five hundred measurements of *a*_13_ in the complex plane with the excitation from source 1 held constant and the output from source 2 set to random phases with constant amplitude.

**Fig. 12:**
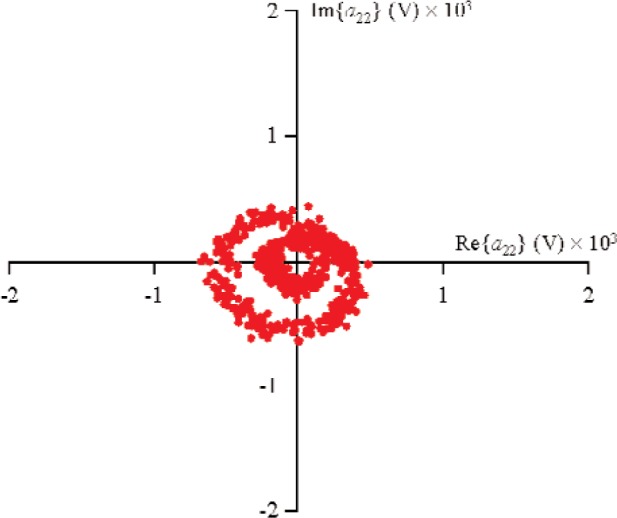
Five hundred measurements of *a*_22_ in the complex plane with the excitation from source 1 held constant and the output from source 2 set to random phases with constant amplitude.

**Fig. 13:**
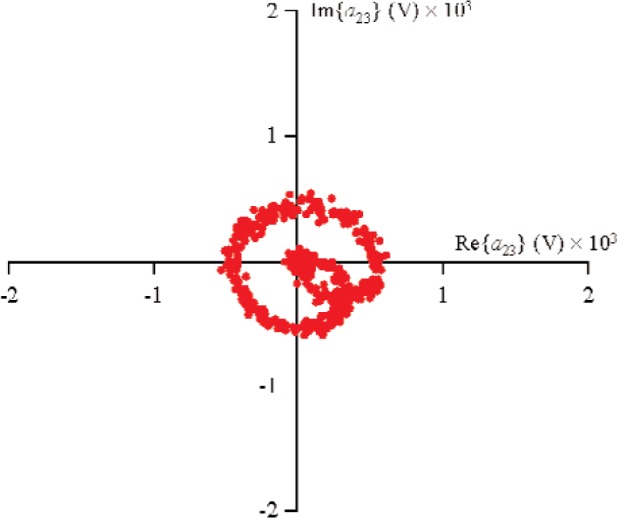
Five hundred measurements of *a*_23_ in the complex plane with the excitation from source 1 held constant and the output from source 2 set to random phases with constant amplitude.

**Fig 14:**
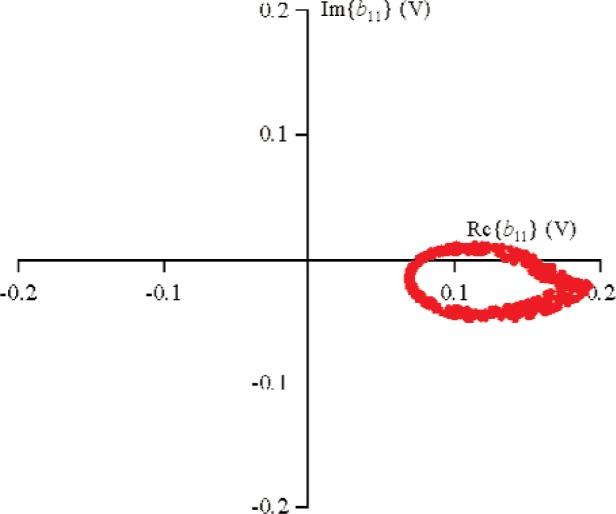
Five hundred measurements of *b*_11_in the complex plane with the excitation from source 1 held constant and the output from source 2 set to random phases with constant amplitude.

**Fig. 15:**
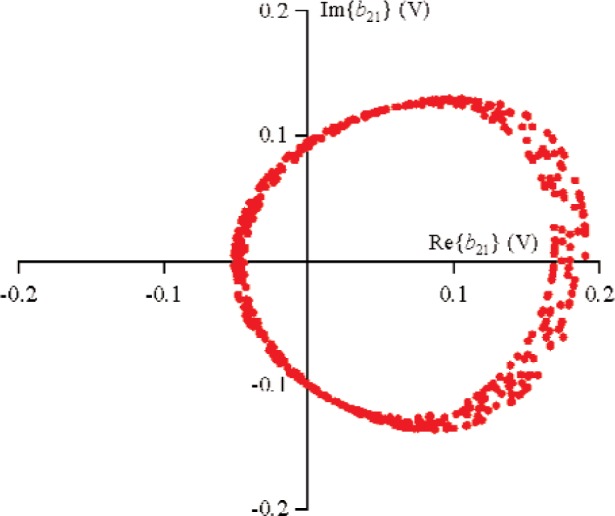
Five hundred measurements of *b*_21_ in the complex plane with the excitation from source 1 held constant and the output from source 2 set to random phases with constant amplitude.

For all measurements, the first source, located at port 1, used a sine-wave excitation of frequency 900 MHz and magnitude |*a*_11_|≈0.178 V (−5 dBm in a 50 Ω environment) at the probe tips. The second source was connected to port 2 and used a sine-wave excitation of frequency 900 MHz and |*a*_21_|≈0.178 V. The diode was forward-biased to +0.2 V through the probe tips. In order to obtain the nonlinear large-signal S-parameters, $S_{11k1}$ and $S_{21k1}$, the excitation from source 1 was held constant, while the phase of source 2 was randomly changed for 500 different measurements that varied slightly in magnitude. Figure 9 plots the resulting measurements of *a*_21_ in the complex plane. The nonlinearities in the test circuit, along with impedance mismatches, created other input components at higher harmonics, as shown in Figs. 10–13 for the second and third harmonics (*a*_12_, *a*_13_, *a*_12_, and *a*_13_). These variations in *a_ij_* allowed us to create an ANN model that could be used to interpolate *b*’s from the measured results for nonzero values of *a_mn_* [(*m*≠1)∧(*n*≠1)], as shown in Figs. 14 and 15 for *b*_11_ and *b*_21_, to the desired values for *a_mn_* [(*m*≠1)∧(*n*≠1)] equal to zero, or alternatively another desired device condition.

### 4.2 Sensitivity Analysis of ANN Models

Data from the 500 measurements were used to develop two ANN models, one for mapping values from the first five harmonics of *a*_1_ and *a*_2_ (*a*_11_, *a*_12_, …, *a*_15_, *a*_21_, *a*_22_, …, *a*_25_) to the first five harmonics of *b*_1_ (*b*_11_, *b*_12_, …, *b*_15_), and the other for mapping values from the first five harmonics of *a*_1_ and *a*_2_ to the first five harmonics of *b*_2_ (*b*_21_, *b*_22_, …, *b*_25_). We performed a sensitivity analysis to determine how many training points, testing points, and hidden neurons are required to adequately train the two ANN models. Tables 2–4 summarize the results for the first model, where we map values from the first five harmonics of *a*_1_ and *a*_2_ to the first five harmonics of *b*_1_, and Tables 5–7 summarize the results for the second model, where we map values from the first five harmonics of *a*_1_ and *a*_2_ to the first five harmonics of *b*_2_.

**Fig. 16:**
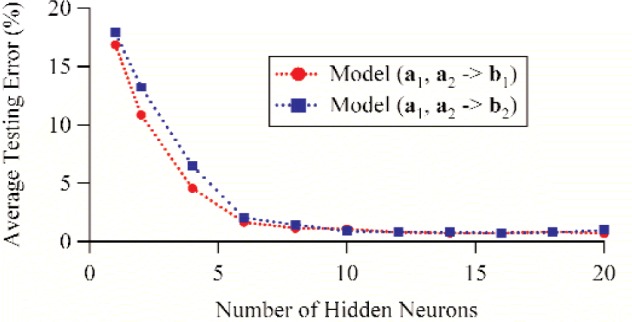
Average testing errors as functions of the number of hidden neurons for ANN models trained to map *a*_1_and *a*_2_ to *b*_1_ and *a*_1_ and *a*_2_ to *b*_2_. The models were developed using 250 training points and verified using 250 testing points.

First, we varied the number of hidden neurons from 1 to 20. All other parameters were held constant. Specifically, the 500 measurements points were divided into 250 training points and 250 testing points, and we used the conjugate gradient method for training. Table 2 lists the average testing errors and correlation coefficients for the models that map *a*_1_ and *a*_2_ to *b*_1_, and Table 5 lists the average testing errors and correlation coefficients for the models that map *a*_1_ and *a*_2_ to *b*_2_. Both mappings show similar trends. The average testing errors decreased with increasing numbers of hidden neurons until around 14 or 16, where the errors were minimized. For more than 16 hidden neurons, the trend reversed and the errors appeared to start increasing again. Figure 16 plots the average testing errors as a function of the number of hidden neurons for both mappings.

**Fig. 17:**
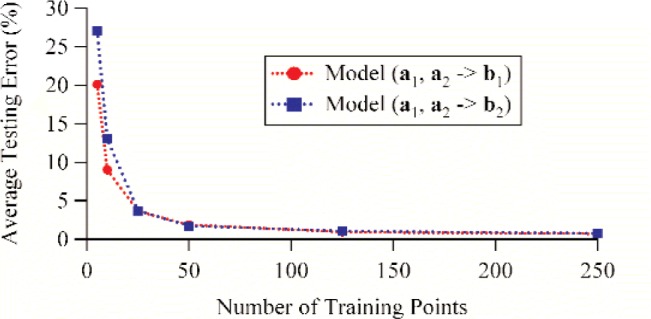
Average testing errors as functions of the number of training points for ANN models trained to map *a*_1_ and *a*_2_ to *b*_1_ and *a*_1_ and *a*_2_ to *b*_2_. The models were developed using 14 hidden neurons and verified using 250 testing points.

Next, we varied the number of training points from 5 to 250. All other parameters were held constant. The number of hidden neurons was set to 14 since we found that to be an ideal number from the previous analysis, and 250 testing points were used for verification. Table 3 lists the average testing errors and correlation coefficients for the models that map *a*_1_ and *a*_2_ to *b*_1_, and Table 6 lists the average testing errors and correlation coefficients for the models that map *a*_1_ and *a*_2_ to *b*_2_. Once again, both mappings showed similar trends. The average testing errors decreased for an increasing number of training points. However, as more and more training points were added, diminishing returns on the testing errors were evident. Figure 17 plots the average testing errors as a function of the number of training points for both mappings.

**Fig. 18:**
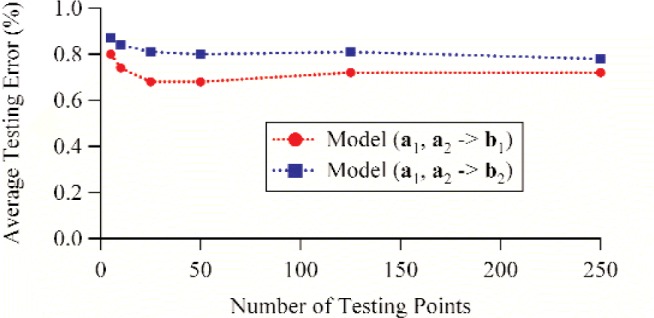
Average testing errors as functions of the number of testing points for ANN models trained to map *a*_1_ and *a*_2_ to *b*_1_ and *a*_1_ and *a*_2_ to *b*_2_. The models were developed using 14 hidden neurons and 250 training points.

Finally, we varied the number of testing points from 5 to 250. All other parameters were held constant. The number of hidden neurons was once again set to 14, and the same 250 training points were used for model development. Table 4 lists the average testing errors and correlation coefficients for the models that map *a*_1_ and *a*_2_ to *b*_1_, and Table 7 lists the average testing errors and correlation coefficients for the models that map *a*_1_ and *a*_2_ to *b*_2_. Both mappings showed that the average testing errors varied little with the number of testing points. Figure 18 plots the average testing errors as a function of the number of testing points for both mappings.

### 4.3 Results and Comparison for Sec. 4

Based on the results of our sensitivity analysis, we decided to use 250 training points and 250 testing points to train and verify the two ANN models. We chose to use 14 hidden neurons for mapping values from the first five harmonics of *a*_1_ and *a*_2_ to the first five harmonics of *b*_1_ and 16 hidden neurons for mapping values from the first five harmonics of *a*_1_ and *a*_2_ to the first five harmonics of *b*_2_. The testing error was 0.72 % for the *b*_1_ model and 0.73 % and for the *b*_2_ model, with respective correlation coefficients of 0.99997 and 0.99992.

**Fig. 19:**
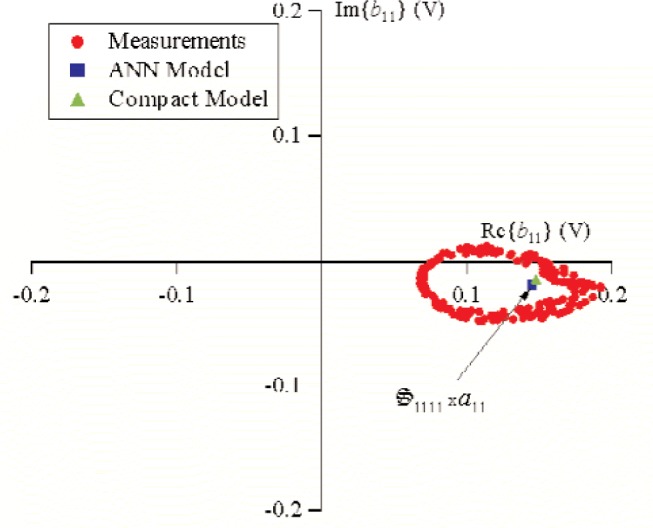
The 250 measurements of *b*_11_ used for training (circles). Values of $S_{1111}\cdot a_{11}$ were determined from the measurement-based ANN model (square) and the harmonic balance simulation using a compact model (triangle).

**Fig. 20:**
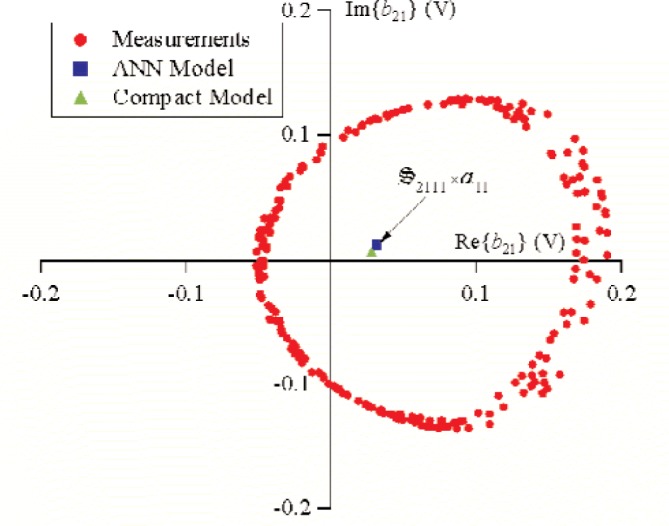
The 250 measurements of *b*_21_ used for training (circles). Values of $S_{2111}\cdot a_{11}$ were determined from the measurement-based ANN model (square) and the harmonic balance simulation using a compact model (triangle).

After the ANN models were developed, the nonlinear large-signal S-parameters, $S_{11k1}$ and $S_{21k1}$ (*k* = 1, 2, …, 5), were obtained by interpolating *b*_1_*_k_* and *b*_2_*_k_* from measured results for nonzero values of *a*_12_, *a*_13_, …, *a*_15_ and *a*_21_, *a*_22_, …, *a*_25_ to the desired values for *a*_12_, *a*_13_, …, *a*_15_ and *a*_21_, *a*_22_, …, *a*_25_ equal to zero. Figure 19 shows the interpolated value of *b*_11_ ( $=S_{1111}\cdot a_{11}$) when *a*_12_, *a*_13_, …, *a*_15_ and *a*_21_, *a*_22_, …, *a*_25_ were set equal to zero, and Fig. 20 shows the interpolated value of *b*_21_ ( $=S_{2111}\cdot a_{11}$) when *a*_12_, *a*_13_, …, *a*_15_ and *a*_21_, *a*_22_, …, *a*_25_ were set equal to zero.

We compared our results to a compact model provided by the manufacturer and simulated in commercial harmonic-balance software to get an independent check on our methodology. Our comparison was accomplished by providing the simulator with the identical biasing conditions on the diode and a stimulus of the same magnitude used in the measurements for *a*_11_ and setting all other *a*’s to zero. Providing the simulated circuit with *a*_11_ of the same magnitude as the measurement should give the same values of *b*_1_*_k_* and *b*_2_*_k_* as the interpolated values of *b*_1_*_k_* ( $=S_{11k1}\cdot a_{11}$) and *b*_2_*_k_* ( $=S_{21k1}\cdot a_{11}$) determined by the ANN models when *a*_12_, *a*_13_, …, *a*_15_ and *a*_21_, *a*_22_, …, *a*_25_ are set equal to zero. Figures 19 and 20 show that the simulated values *b*_11_ and *b*_21_ agree with those determined from the measurement-based ANN models.

Quantitatively, the differences between the ANN and equivalent-circuit models are shown in Table 8.

### 4.4 Summary of Sec. 4

We described a method of extracting nonlinear large-signal S-parameters, using an NVNA equipped with isolators and a second source. First, we showed how multiple measurements of a nonlinear circuit could be used to train artificial neural networks. Then, we extracted the desired S-parameters by interpolating the ANN models for all *a*’s equal to zero other than *a*_11_. We checked our approach by comparing our results to a compact model simulated in commercial harmonic-balance software, and showed that the two methods agree well.

We also performed a sensitivity analysis on the ANN networks, and discovered the following: (1) The average testing error decreases for an increasing number of training points. However, as more and more training points are added, diminishing returns on the testing errors are evident. (2) As the number of hidden neurons are increased, the average testing error decreases until around 14 hidden neurons at which point more hidden neurons have no benefit and can actually lead to increases in testing error. (3) The number of testing points does not drastically affect the testing error. In fact, no more than 25 testing points are needed for the models tested.

## 5. Overall Summary

In this paper, we introduced nonlinear large-signal scattering parameters representing a new type of frequency-domain mapping that relates incident and reflected signals. Unlike classical *S*-parameters, nonlinear large-signal S-parameters take harmonic content into account and depend on the signal magnitudes. First, we presented a general form of nonlinear large-signal S-parameters and showed that they reduce to classic *S*-parameters in the absence of nonlinearities. We also introduced nonlinear large-signal impedance ( Z) and admittance ( D) parameters, and presented equations that relate the different representations. Next, we considered two simplified cases of a one-port network and a two-port network, each with a single-tone excitation. For the one-port network, we showed that the equation relating S and Z reduces to the same well-known equation for the linear case, assuming no power is transferred in the form of frequency down-conversion. For the two-port case, we extracted input reflection coefficients and forward transmission coefficients, which can be useful for designing circuits such as amplifiers and frequency multipliers. In addition, we derived a quasi-linear approximation of the output reflection coefficient under normal operating conditions. These three two-port parameters allow a designer to “see” application-specific engineering figures of merit that are similar to what he or she is accustomed to in the linear world.

Next, we illustrated how nonlinear large-signal S-parameters can be used as a tool in the design process of a single-diode 1 GHz frequency-doubler. Specifically, we used $S_{1111}$ to determine the input matching network, $S_{2222}$ to determine the output matching network, and $S_{11k1},\;S_{21k1}$ (for *k* = 1 to 6), and $G_{2}$ to quantify the performance of the circuit at each stage. By the final stage of the design, we had created a doubler with an overall power gain of −9.56 dB, a value not far from the maximum possible predicted value of −9 dB.

For the case where a nonlinear model is not readily available, we described a method of extracting nonlinear large-signal S-parameters, using an NVNA equipped with isolators and a second source. First, we showed how multiple measurements of a nonlinear circuit could be used to train artificial neural networks. Then, we extracted the desired S-parameters by interpolating the ANN models for all *a*’s equal to zero other than *a*_11_. We checked our approach by comparing our results to a compact model simulated in commercial harmonic-balance software, and showed that the two methods agree well. We also performed a sensitivity analysis on the ANN networks, and discovered the following: (1) The average testing error decreases for an increasing number of training points. However, as more and more training points are added, diminishing returns on the testing errors are evident. (2) As the number of hidden neurons are increased, the average testing error decreases until around 14 hidden neurons, at which point more hidden neurons have no benefit and can actually lead to increases in testing error. (3) The number of testing points does not drastically affect the testing error. In fact, no more than 25 testing points are needed for the models tested.

## Figures and Tables

**Fig. 21:**
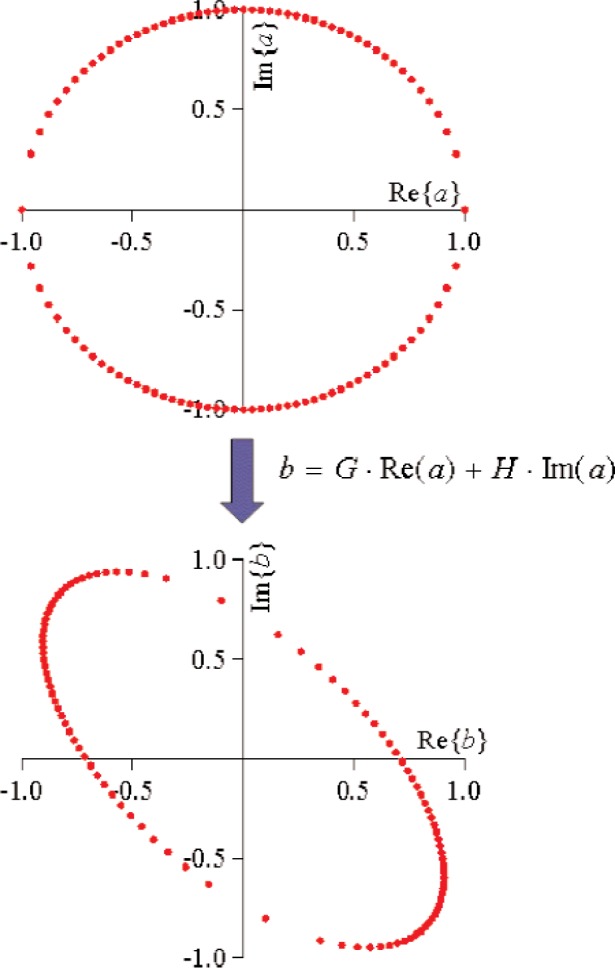
*G_kpij_* and *H_kpij_* serve to map *a_ij_* circles centered at zero to *b_kp_* ellipses with variable axes also centered at zero, neglecting *F_kp_* for illustrative purposes.

**Fig. 22:**
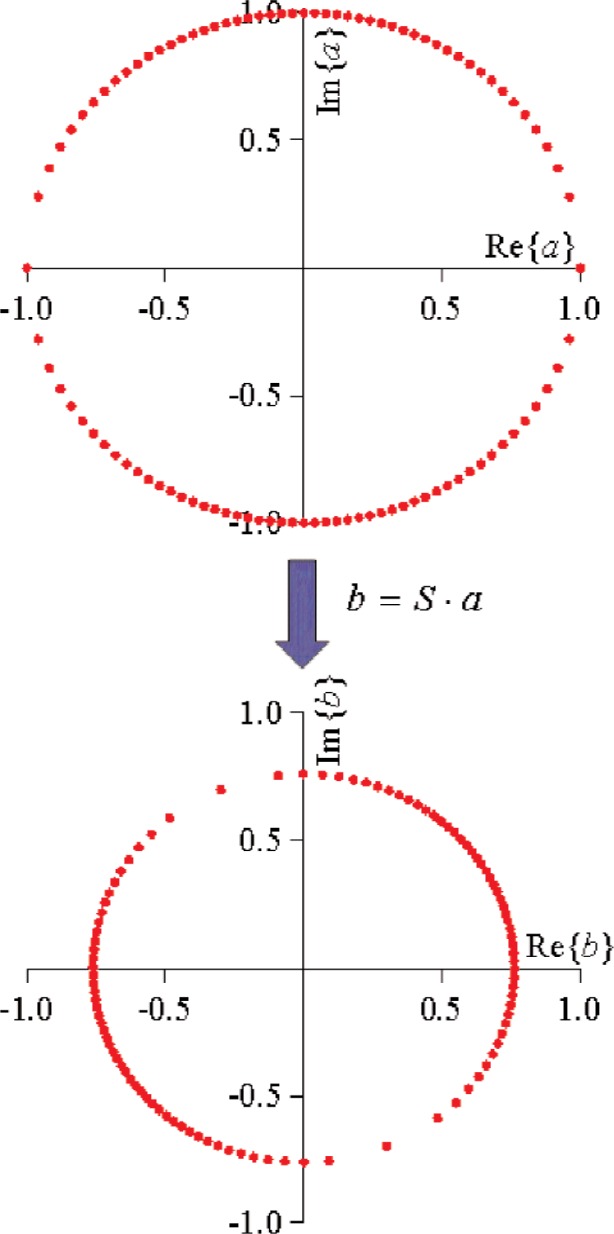
If $S_{ijkl}$ is a complex constant for a given bias and fundamental drive level, it has the limitation that it can only map circles into circles.

**Table 1:** Simulated values for $S_{1111}-S_{1161},\;S_{2111}-S_{2161},\;G_{2}$ and $G_{2}/G$ for each of the design stages of the diode frequency doubler

Quantity	Diode only	Diode w/ 1, 2 GHz filters	Diode w/ 1, 2 GHz filters input match	Diode w/ 1, 2 GHz filters, input & output match	Diode w/ 1, 2 GHz filters, input & output match opt.	Diode w/ 1, 2, 4, 6 GHz filters input & output match opt.
$\left|S_{1111}\right|$	0.464	0.569	9.4×10^−2^	8.7×10^−2^	6.0×10^−3^	2.1×10^−4^
$\left|S_{1121}\right|$	0.170	1.3×10^−5^	8.8×10^−6^	8.0×10^−6^	9.5×10^−6^	9.9×10^−6^
$\left|S_{1131}\right|$	3.2×10^−2^	4.9×10^−3^	4.0×10^−3^	1.4×10^−2^	1.1×10^−2^	2.2×10^−2^
$\left|S_{1141}\right|$	2.4×10^−2^	3.5×10^−2^	3.7×10^−2^	2.4×10^−2^	2.8×10^−2^	5.1×10^−2^
$\left|S_{1151}\right|$	1.7×10^−2^	1.1×10^−2^	1.1×10^−2^	1.9×10^−3^	2.3×10^−3^	2.5×10^−3^
$\left|S_{1161}\right|$	3.9×10^−3^	1.0×10^−6^	1.0×10^−6^	9.7×10^−7^	1.1×10^−6^	2.0×10^−6^
$\left|S_{2111}\right|$	0.536	3.3×10^−5^	4.0×10^−5^	4.0×10^−5^	4.0×10^−5^	5.0×10^−5^
$\left|S_{2121}\right|$	0.170	0.268	0.326	0.328	0.331	0.332
$\left|S_{2131}\right|$	3.2×10^−2^	3.5×10^−7^	3.3×10^−7^	1.5×10^−6^	1.1×10^−6^	1.7×10^−7^
$\left|S_{2141}\right|$	2.4×10^−2^	3.5×10^−2^	4.5×10^−2^	4.1×10^−2^	4.0×10^−2^	1.4×10^−6^
$\left|S_{2151}\right|$	1.7×10^−2^	7.6×10^−7^	1.1×10^−6^	2.5×10^−6^	2.3×10^−6^	3.0×10^−6^
|S2161|	3.9×10^−3^	2.0×10^−2^	2.5×10^−2^	2.6×10^−2^	2.9×10^−2^	2.7×10^−6^
$G_{2}$(dB)	−14.16	−9.73	−9.69	−9.65	−9.60	−9.56
$G_{2}/G$	0.091	0.978	0.976	0.979	0.978	0.999

**Table 2:** Average testing errors and correlation coefficients as functions of the number of hidden neurons for ANN models trained to map values from the first five harmonics of *a*_1_ and *a*_2_ to the first five harmonics of *b*_1_. All models were developed using 250 training points and verified using 250 testing points

Hidden neurons	Average testing error (%)	Correlation Coefficient
1	16.86	0.94814
2	10.84	0.98896
4	4.56	0.99715
6	1.66	0.99971
8	1.15	0.99989
10	1.08	0.99991
12	0.80	0.99996
14	0.72	0.99997
16	0.72	0.99997
18	0.84	0.99996
20	0.70	0.99997

**Table 3:** Average testing errors and correlation coefficients as functions of the number of training points for ANN models trained to map values from the first five harmonics of *a*_1_ and *a*_2_ to the first five harmonics of *b*_1_. All models were developed using 14 hidden neurons and verified using 250 testing points

Training points	Average testing error (%)	Correlation Coefficient
5	20.10	0.96764
10	9.01	0.99556
25	3.64	0.99891
50	1.91	0.99979
125	0.95	0.99995
250	0.72	0.99997

**Table 4:** Average testing errors and correlation coefficients as functions of the number of testing points for ANN models trained to map values from the first five harmonics of *a*_1_ and *a*_2_ to the first five harmonics of *b*_1_. All models were developed using 250 training points and 14 hidden neurons

Testing points	Average testing error (%)	Correlation Coefficient
5	0.80	0.99998
10	0.74	0.99997
25	0.68	0.99998
50	0.68	0.99998
125	0.72	0.99997
250	0.72	0.99997

**Table 5:** Average testing errors and correlation coefficients as functions of the number of hidden neurons for ANN models trained to map values from the first five harmonics of *a*_1_ and *a*_2_ to the first five harmonics of *b*_2_. All models were developed using 250 training points and verified using 250 testing points

Hidden neurons	Average testing error (%)	Correlation Coefficient
1	17.88	0.74320
2	13.22	0.91161
4	6.48	0.96659
6	2.04	0.99893
8	1.43	0.99951
10	0.90	0.99985
12	0.82	0.99989
14	0.78	0.99989
16	0.73	0.99992
18	0.78	0.99988
20	0.99	0.99983

**Table 6:** Average testing errors and correlation coefficients as functions of the number of training points for ANN models trained to map values from the first five harmonics of *a*_1_ and *a*_2_ to the first five harmonics of *b*_2_. All models were developed using 14 hidden neurons and verified using 250 testing points

Training points	Average testing error (%)	Correlation Coefficient
5	27.08	0.50237
10	12.99	0.91962
25	3.72	0.99628
50	1.75	0.99940
125	1.09	0.99978
250	0.78	0.99989

**Table 7:** Average testing errors and correlation coefficients as functions of the number of testing points for ANN models trained to map values from the first five harmonics of *a*_1_ and *a*_2_ to the first five harmonics of *b*_2_. All models were developed using 250 training points and 14 hidden neurons

Testing points	Average testing error (%)	Correlation Coefficient
5	0.87	0.99995
10	0.84	0.99993
25	0.81	0.99988
50	0.80	0.99989
125	0.81	0.99988
250	0.78	0.99989

**Table 8:** Differences between the measurement-based, ANN-modeled results and the compact model simulated in commercial harmonic-balance software

Quantity	Difference (%)	Difference (dBV)
$S_{1111}$	3.38	–44.5
$S_{1121}$	1.23	–53.3
$S_{1131}$	3.29	–44.8
$S_{1141}$	0.40	–63.1
$S_{1151}$	1.67	–50.6
$S_{2111}$	3.95	–43.2
$S_{2121}$	7.15	–38.0
$S_{2131}$	5.93	–39.6
$S_{2141}$	0.72	–57.9
$S_{2151}$	0.85	–56.5

## 6. Appendix A. Comparing Nonlinear Large-Signal S-Parameters With Nonlinear Scattering Functions

Here, we compare the nonlinear large-signal S-parameters, introduced in this paper, to another form of nonlinear mapping, known as nonlinear scattering functions, introduced by Verspecht [6–7].

For a two-port nonlinear device, excited by a single-tone signal, and assuming all harmonic signals are relatively small compared to the fundamental signals, Verspecht defines nonlinear scattering functions as

$$b_{kp}=F_{kp}+\sum_{\begin{array}{l} i=1,2\\ j=2,\ldots ,M \end{array}}G_{kpij}\text{Re}(a_{ij})+\sum_{\begin{array}{l} i=1,2\\ j=2,\ldots ,M \end{array}}H_{kpij}\text{Im}(a_{ij}),$$ (59)

where *a_ij_* and *b_kp_* represent the wave variables proportional to the incoming and outgoing waves, respectively, and *M* refers to the number of harmonics being taken into account. *F_kp_*, *G_kpij_*, and *H_kpij_* are functions of the fundamental components Re(*a*_11_), Re(*a*_21_), and Im(*a*_21_). The imaginary component of *a*_11_ is omitted, with the assumption that the wave variables are phase referenced such that the phase of *a*_11_ is set to zero. *F_kp_*, *G_kpij_*, and *H_kpij_* are assumed complex constants for a given bias and fundamental drive condition. Note that these three terms do not depend upon the higher harmonic signal levels. With the *a_ij_* wave variables split into real and imaginary components, *G_kpij_* and *H_kpij_* serve to map *a_ij_* circles centered at zero to *b_kp_* ellipses with variable axes also centered at zero, as shown in Fig. 21. The *F_kp_* terms translate the ellipses about the complex plane.

For illustrative purposes, let us consider *b_11_*, taking into account the first three harmonics. Doing this, Eq. (59) reduces to

$$b_{11}=F_{11}+\sum_{\begin{array}{l} i=1,2\\ j=2,3 \end{array}}G_{11ij}\text{Re}(a_{ij})+\sum_{\begin{array}{l} i=1,2\\ j=2,3 \end{array}}H_{11ij}\text{Im}(a_{ij})$$ (60)

or

$$\begin{array}{c} b_{11}=F_{11}+G_{1112}\text{Re}(a_{12})+H_{1112}\text{Im}(a_{12})\\ +G_{1113}\text{Re}(a_{13})+H_{1113}\text{Im}(a_{13})\\ +G_{1122}\text{Re}(a_{22})+H_{1122}\text{Im}(a_{22})\\ +G_{1123}\text{Re}(a_{23})+H_{1123}\text{Im}(a_{23}). \end{array}$$ (61)

If we now consider the nonlinear large-signal S-parameter representation for *b*_11_, once again assuming a two-port network and taking into account the first three harmonics, we have

$$b_{11}=\sum_{\begin{array}{l} j=1,2\\ l=2,3 \end{array}}S_{1j1l}a_{jl}$$ (62)

or

$$\begin{array}{l} b_{11}=S_{1111}a_{11}+S_{1112}a_{12}+S_{1113}a_{13}\\ \;+S_{1211}a_{21}+S_{1212}a_{22}+S_{1213}a_{23}. \end{array}$$ (63)

Here, $S_{ijkl}$ are functions of all of the harmonics, not just the fundamental terms. So for any change in any *a_jl_*, a new set of $S_{ijkl}$ will need to be determined. Separating the real and imaginary components of the *a*’s, we can express eq. (63) as

$$\begin{array}{l} b_{11}=S_{1111}\text{Re}(a_{11})+S_{1112}[\text{Re}(a_{12})+j\text{Im}(a_{12})]\\ \;+S_{1113}[\text{Re}(a_{13})+j\text{Im}(a_{13})]+S_{1211}[\text{Re}(a_{21})+j\text{Im}(a_{21})]\\ \;+S_{1212}[\text{Re}(a_{22})+j\text{Im}(a_{22})]+S_{1213}[\text{Re}(a_{23})+j\text{Im}(a_{23})]. \end{array}$$ (64)

Once again, the imaginary component of *a*_11_ is omitted, with the phase reference such that the phase of *a*_11_ is set to zero.

We can now equate the nonlinear large-signal S-parameters of Eq. (64) to the nonlinear scattering functions of Eq. (61), with the understanding that this is only generally valid for the special case when the nonlinear large-signal S-parameters are constant for a given bias and fundamental drive level, like *F_kp_*, *G_kpij_*, and *H_kpij_* are defined. Normally, however, the nonlinear large-signal S-parameters depend upon the higher harmonics as well as on the bias and fundamental drive level. The implication of this special case will be discussed shortly, after Eqs. (61) and (64) are equated. Equating the corresponding real and imaginary components of the *a* wave variables in Eqs. (61) and (64) gives

$$F_{11}=S_{1111}\text{Re}(a_{11})+S_{1211}a_{21}.$$ (65)

Additionally,

$$S_{1112}=G_{1112};\;jS_{1112}=H_{1112},$$ (66)

$$S_{1113}=G_{1113};\;jS_{1113}=H_{1113},$$ (67)

$$S_{1212}=G_{1122};\;jS_{1212}=H_{1122},$$ (68)

and

$$S_{1213}=G_{1123};\;jS_{1213}=H_{1123}.$$ (69)

Equations (66)–(69) imply

$$G_{1112}=-jH_{1112};G_{1113}=-jH_{1113};G_{1122}=-jH_{1122};G_{1123}=-jH_{1123},$$ (70)

which means

$$\text{Re}(G_{kpij})=\text{Im}(H_{kpij});{\text{Re}(H}_{kpij})=-\text{Im}(G_{kpij}).$$ (71)

Equation (71) satisfies the conditions of the Cauchy-Riemann equations [15],

$$\frac{\partial [\text{Re}(b_{kp})]}{\partial [\text{Re}(a_{ij})]}=\frac{\partial [\text{Im}(b_{kp})]}{\partial [\text{Im}(a_{ij})]};\frac{\partial [\text{Re}(b_{kp})]}{\partial [\text{Im}(a_{ij})]}=-\frac{\partial [\text{Im}(b_{kp})]}{\partial [\text{Re}(a_{ij})]},$$ (72)

which implies *b_kp_* must be an analytic function of *a_ij_*. A complex-valued function is said to be analytic on an open set *W* if it has a derivative at every point of *W*. This is generally true only when *b_kp_* is a linear function of *a_ij_*. Thus, equating the nonlinear large-signal S-parameters with the nonlinear scattering functions is generally valid only in the small-signal, linear case.

As we mentioned earlier, Eqs. (65)–(70) are only generally valid in the special case when the nonlinear large-signal S-parameters are constant for a given bias and fundamental drive level, like *F_kp_*, *G_kpij_*, and *H_kpij_* are defined. Since this is not generally true, the formulations for nonlinear large-signal S-parameters and nonlinear scattering functions are not equivalent.

We can draw a few important conclusions, however, after attempting to equate the two formulations. First, if *G_kpij_* and *H_kpij_* are allowed to be functions of higher harmonics, then only one of them, either *G_kpij_* or *H_kpij_*, or equivalently $S_{ijkl}$, is required since Eq. (70) shows that they are not independent. Second, if the nonlinear large-signal S-parameters are complex constants for a given bias and fundamental drive level and are not functions of the higher harmonics, the parameters have the limitation that they cannot map circles into ellipses, but rather can only map circles into circles, as shown in Figure 22. This is because $S_{ijkl}$ is a single, complex constant rather than a pair of independent complex constants such as *G_kpij_* and *H_kpij_*. Thus, if $S_{ijkl}$ is not dependent upon higher harmonics, it acts like a linear *S*-parameter.

We have shown above that the two formulations are not equivalent. Nonlinear large-signal S-parameters are more general than the nonlinear scattering functions, which are useful in approximating a specific class of nonlinearity in a more compact form. Nonlinear large-signal S-parameters have the advantage of being able to map circles into any arbitrary shape, rather than being limited to ellipses.
